# Innovative Biocatalysts as Tools to Detect and Inactivate Nerve Agents

**DOI:** 10.1038/s41598-018-31751-5

**Published:** 2018-09-13

**Authors:** Elena Porzio, Francesca Bettazzi, Luigi Mandrich, Immacolata Del Giudice, Odile F. Restaino, Serena Laschi, Ferdinando Febbraio, Valentina De Luca, Maria G. Borzacchiello, Teresa M. Carusone, Franz Worek, Antonio Pisanti, Piero Porcaro, Chiara Schiraldi, Mario De Rosa, Ilaria Palchetti, Giuseppe Manco

**Affiliations:** 1 Institute of Protein Biochemistry, National Research Council of Italy, Naples, Italy; 2 Department of Chemistry, University of Florence, Sesto Fiorentino (FI), Italy; 3 University of Campania “Luigi Vanvitelli”, Naples, Italy; 4 Ecobioservices and Research srl, Modica (RG), Italy; 5 Bundeswehr Institute of Pharmacology and Toxicology, Munich, Germany; 6 Tecno Bios srl, Apollosa (BN), Italy

## Abstract

Pesticides and warfare nerve agents are frequently organophosphates (OPs) or related compounds. Their acute toxicity highlighted more than ever the need to explore applicable strategies for the sensing, decontamination and/or detoxification of these compounds. Herein, we report the use of two different thermostable enzyme families capable to detect and inactivate OPs. In particular, mutants of carboxylesterase-2 from *Alicyclobacillus acidocaldarius* and of phosphotriesterase-like lactonases from *Sulfolobus solfataricus* and *Sulfolobus acidocaldarius*, have been selected and assembled in an optimized format for the development of an electrochemical biosensor and a decontamination formulation, respectively. The features of the developed tools have been tested in an *ad-hoc* fabricated chamber, to mimic an alarming situation of exposure to a nerve agent. Choosing ethyl-paraoxon as nerve agent simulant, a limit of detection (LOD) of 0.4 nM, after 5 s of exposure time was obtained. Furthermore, an optimized enzymatic formulation was used for a fast and efficient environmental detoxification (>99%) of the nebulized nerve agent simulants in the air and on surfaces. Crucial, large-scale experiments have been possible thanks to production of grams amounts of pure (>90%) enzymes.

## Introduction

Pesticides and warfare nerve agents are frequently organophosphates (OPs) or related compounds (e.g. organophosphonates). Synthetic OPs are highly toxic chemicals that originally developed for crop protection quickly enthused their exploitation in the more lethal chemical warfare agents (CWAs)^1^. Their acute toxicity and the recent dramatic events in Syria highlighted more than ever the need to explore applicable strategies for the sensing, decontamination and/or detoxification of these and related compounds^2^. Many countries have accumulated huge stocks of them starting from the World War 2 until the cold war^3^. In 1993, an International Convention, signed by 192 countries, called for stopping the development of these weapons, and planned for the destruction of existing stocks within the deadline of 2007. The deadline has been eventually postponed to 2023 since some states possessing the largest arsenals were not able to meet the date partly because of the lack of low-cost, rapid, environmental-friendly and safe solutions to destroy them^4,5^. Large stocks, therefore still exist today. They have been and are still used in regional conflicts such as the Iran-Iraq war (1980–1988)^6^, and the recent civil war in Syria^2^. Due to their strong emotional negative effect on people, they have also been used as terrorist weapons^7^.

As far as permitted uses are concerned, OP pesticides constitute the largest class of worldwide-employed insecticides (i.e., parathion, malathion, etc.). According to the U.S. Environmental Protection Agency 20 million pounds of them have been used in USA in 2012 and, more than for CWA, huge stocks of “obsolete pesticides” are present worldwide, particularly in the third world countries that need to be disposed of ^8^. OP pesticide poisoning has become a global health problem, intoxicating millions of people if one consider acute and chronic unintentional intoxications. In fact, a conservative estimate based on recent data indicates that there were approximately 110,000 pesticide self-poisoning deaths each year from 2010 to 2014^9^.

Both OP pesticides and nerve gases are neurotoxins because they inhibit the key enzyme of the nervous system, acetylcholinesterase (AChE), which regulates the turnover of the neurotransmitter acetylcholine at the synaptic transmission^1^. The covalent inhibition of AChE results in the accumulation of acetylcholine at the neuron-neuron or neuron-muscle junctions leading to various clinical complications and, ultimately, to death^10^.

Many toxicological studies have also revealed the genotoxic and carcinogenetic effects of OP pesticides; they are able to cause gene mutations, chromosomal aberrations, and DNA damage in mammals^11^, and alteration of semen quality in human^12^. Recently, a study reported on Lancet Oncology (2015)^13^, identified tetrachlorvinphos, parathion, malathion, diazinon and glyphosate as likely carcinogens in mammals. Some of them are already in the list of banned pesticides in EU.

The recent events in Middle East with nerve agent Sarin^2^, solicit timely solutions for immediate personal protection, recovery of equipment and buildings, containment of agent spills, bulk destruction of chemical weapon stockpiles^5^. Such solutions could be of benefit also for the problems posed by less toxic pesticides.

Methods that can detoxify both materials and personnel are under investigations^14^.

Solid heterogeneous materials such as modified activated carbon or metal oxides exhibit many desirable characteristics for the destruction of CWAs for fabrics and protection tools (masks, suites, filters for air conditioning systems, wipes with immobilised enzymes)^15–18^. Unfortunately, the catalytic activity of these materials is still too low to be of practical use in the short run. In fact, existing drawbacks are slow degradation kinetics, deactivation of the active site, low adsorptive capacities, low effective active site loadings, which offer significant room for improvement^18^. Efficient chemical methods including hypochlorite and sodium hydroxide treatments have emerged for the cheap environmental detoxification of OPs. These methods usually involve harsh chemical conditions and are not compatible with the care of personnel or sensitive materials^14^. Biocatalysts in free (or immobilized) form represent a good alternative. Recent investigations are focusing on the exploitation of OP-sensing^19,20^, and OP-degrading enzymes^21^. The use of enzyme technology is appealing because it offers efficiency and specificity under mild conditions, is non-corrosive, is safe and eco-friendly and allows human therapy^1,22–24^. Finally, by reducing process time, biocatalysis is energy and water saving compared with chemical processes^25,26^. Whereas high efficiency, stability and production costs of enzymes may be an issue, the discovery of highly thermostable carboxylesterases and OPs degrading enzymes and their successful engineering to improve their properties^27,28^, is expected to turn them into competitive and economically attractive OP biosensors and biodetoxicants. Furthermore, enzymes can be easily immobilized on different matrices including foams, with extra advantages (e.g. recycling)^29,30^.

Along the vein of this research, here we report the probing of free and immobilised thermostable enzymes on different materials and surfaces. In particular, mutants of carboxylesterase-2 from *Alicyclobacillus acidocaldarius* (EST2) and of phosphotriesterase-like lactonases (PLL) from *Sulfolobus solfataricus* and *Sulfolobus acidocaldarius*, have been selected and assembled in an optimized format for the development of an electrochemical biosensor and a decontamination formulation, respectively. Furthermore, the building, testing and validation of an integrated system based on a highly responsive biosensor and a hydraulic/informatics setup that controls the nebulization of an enzymatic formulation, for the fast (seconds) and efficient (>99%) environmental detoxification of nerve agent simulants in the air and surfaces is also described. Crucial large-scale experiments were possible thanks to production of gram amounts of pure (>90%) enzymes.

## Results and Discussion

### Selection of thermostable biocatalysts

Key biological components of the systems analyzed here are thermostable members of two families of enzymes that we evolved *in vitro*, produced at high level from *E*. *coli* and employed for the set-up of biosensors and enzyme formulations. The optimization of formulations was required as to permit direct environmental application *via* nebulization with the aim to inactivate OPs in the airstream and on different surfaces and materials.

EST2 is a member of the hormone sensitive lipase family^31^, and shows as the human AChE^1^, a very high sensitivity toward covalent inhibition by OP pesticide like paraoxon (POX)^20^. POX has been used here also as simulant of the more deadly nerve gases (sarin, soman, tabun) both as inhibitor or as substrate. Although differences have been pointed out by some researchers, still POX is largely used as nerve agent model by many scientists^32^, especially in proof-of-concept research, owing to the difficulty in working with real nerve agents. This kind of test is usually performed with mimic compounds, which have much easier handling characteristics.

**Figure 1:**
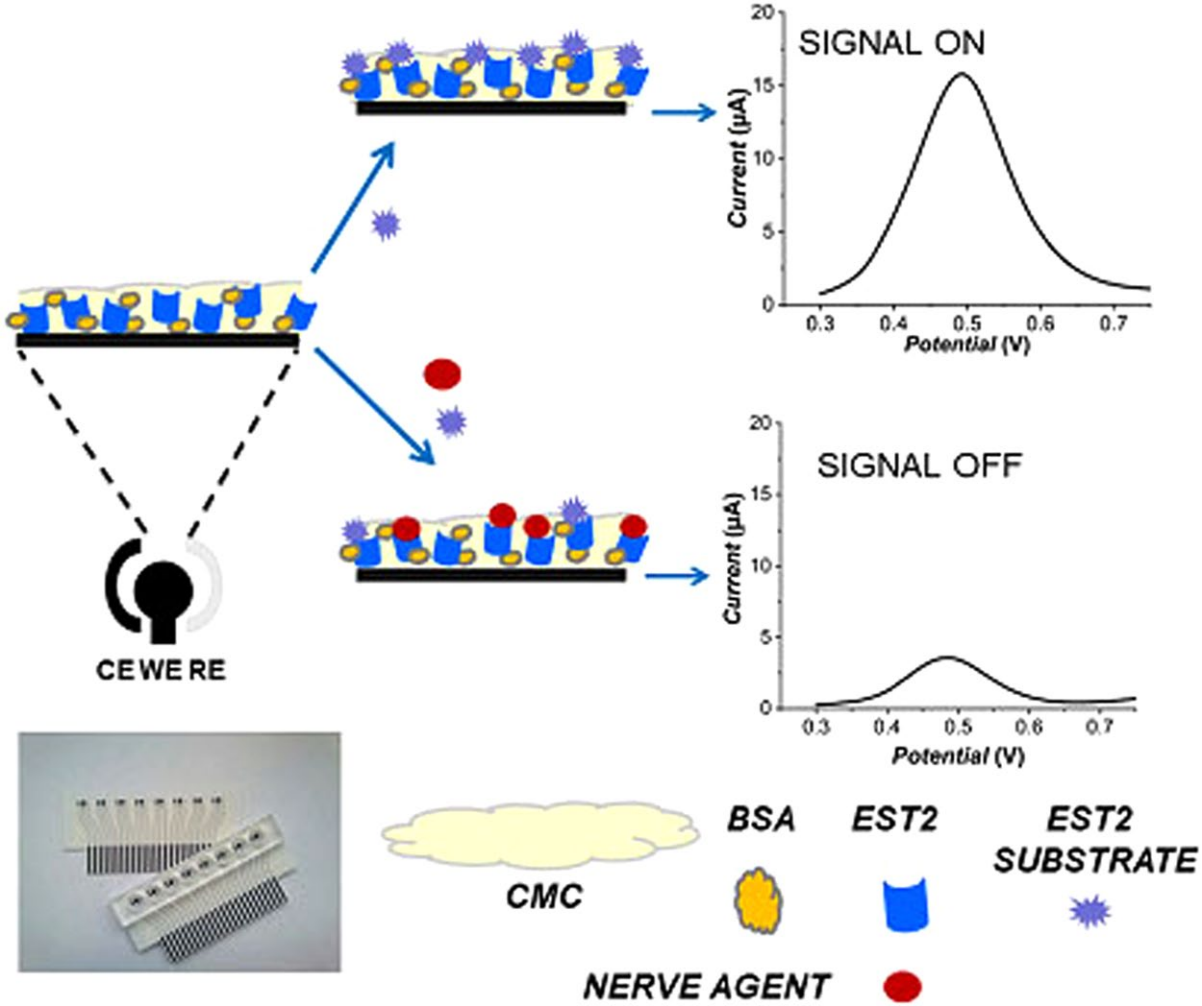
Scheme of the assay. The biosensor is obtained by modification of the carbon electrode surface with the enzymatic layer (EST2 mutant, BSA and CMC). Upon the addition of NaAc, the electroactive product (2-naphthol) is oxidized at the electrode and a peak current is recorded in DPV. If a nerve agent is present, the enzyme will be inhibited, and a decrease of the peak current is observed. Sensor array used for developing the biosensing MEP, without and with the plastic wells.

The monitoring over time of the residual activity of the enzyme after encounter with the inhibitor gives the indirect measurement of its concentration^33,34^. In a previous report we exploited wild type EST2 for biosensing application^33^. In the present study, we focused on the double mutant K42R/K61R (positions shown in the EST2 structure Fig. S1a; see supplementary text and ref.^35^) to exploit its higher stability than the wild type (Fig. S1b,c). The catalytic activity, evaluated spectrophotometrically using *p*-NP-hexanoate as substrate, was found to be 10,500 μmol/min/mg. This value was better than those of the wild type (7,500 μmol/min/mg). The high turnover number of the mutant permits the activity measurement to be made in a short time, and accordingly to obtain a rapid response of the biosensor. Thus, the mutant, purified as described^35^, was used in the assembly of an electrochemical biosensor (Fig. 1). Furthermore, recently we discovered the new family of PLLs^36,37^. Within the family, particular attention was given to the thermostable members from *S*. *solfataricus* (*Sso*) and *S*. *acidocaldarius* (*Sac*), which were endowed with promiscuous yet moderately high phosphotriesterase (PTE) activity against OPs^38^. The thermostable enzymes are the favorite choice of protein engineers when attempting *in vitro* molecular evolution approaches with the final aim to obtain catalysts with unique features such as stability to different harsh conditions combined with high proficiency against selected substrates^39^. Old and newly produced variants of PLLs have been analyzed and after a process in which we first evaluated isolated variants and then some of their combinations (see below), we finally come up with a mixture of 3 or 4 enzymes dubbed ENZYMIX3 or ENZYMIX4 respectively. Mixes were made of the wild types *Sac* PLL (*Sac*Pox)^40^ and *Sso* PLL (*Sso*Pox)^41^, the single mutant *Sso*PoxTrp263Phe (SsoW263F)^27^ and the triple mutant *Sso*Pox Cys258Leu/Ile261Phe/Trp263Ala (*Sso*3Mut)^28^. The ENZYMIX was selected following a careful analysis of the different substrate specificities (Table S1) and enzyme stability under different conditions, such as the presence of solvents and detergents (Fig. S2 and SI) of the single enzymes. The goal was to get hold of an enzyme formulation able to destroy promptly and entirely a mixture of three different toxic OPs namely POX, methyl-paraoxon (MPOX) and methylparathion (MPTON), in buffered aqueous matrices and on different materials and surfaces. This mixture of compounds is used here as less toxic model of a *“live”* nerve agents solution (NAS), for routine analyses.

### Electrochemical biosensor

To build an efficient integrated system able to sense and detoxify OPs in a closed space, we first focused on the sensing component. Figure 1 shows the operating principle for the measurement of OPs using the proposed electrochemical biosensor.

The biosensor incorporates a multichannel electrochemical platform (MEP) based on screen-printing technology allowing mass production at low cost. Specifically, the MEP consists of eight independent, compact, electrochemical cells (Fig. 1), with a carbon working electrode (WE), a silver *pseudo-reference* electrode (RE), and a graphite counter-electrode (CE). Each electrochemical cell was used as disposable to avoid fouling of the active area of the WE and thus the need of electrode regeneration. Therefore, the biosensor itself can be used as a disposable tool for time-specific monitoring over short lengths of time.

To convert the MEP in biosensor, the enzyme (K42R/K61R mutant) was immobilized by cross-linking with carboxymethylcellulose (CMC) and bovine serum albumin (BSA) onto the surface of the WE (Fig. 1). The immobilized enzyme converts 2-Naphtylacetate (NaAc) to its electroactive product, 2-naphthol (2-Na), which is detected by Differential Pulse Voltammetry (DPV) measurements. The peak current generated during the electrochemical oxidation of 2-Na constitutes the analytical response. In the presence of OPs, the enzyme is inhibited leading to a reduced production of 2-Na and hence a lower anodic peak current. This decrease is proportional to the OP concentration.

An inhibition percentage (I%; Eq. 1) can be calculated according to the following formula:

1 $$I \% ={100}^{\ast }({I}_{0}-{I}_{x})/{I}_{0}$$

where I_0_ is the peak current value in the absence of the nerve agent and I_x_ the current obtained after exposure to the nerve agent.

#### Analytical Performances of the electrochemical biosensor

**Figure 2:**
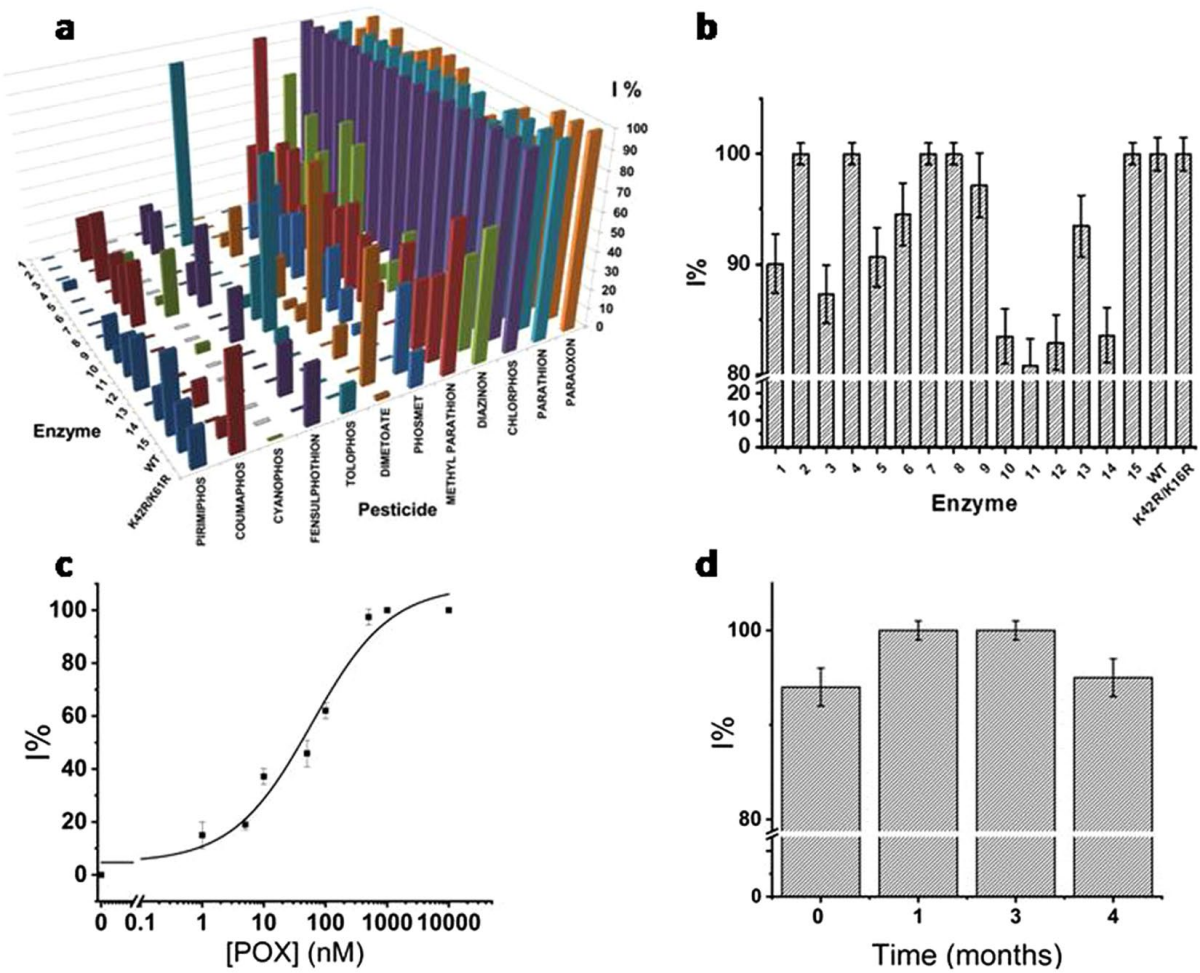
Development of the electrochemical biosensing assay. (**a**) Inhibitory effect of different toxicants on EST2 wt, K42R/K61R and other mutants. For presentation clarity, the effect of POX on the different mutants is also shown in the next panel (**b**); Different toxicants were diluted to 1 μM in PB containing N-bromosuccinimide (NBS) in a ratio of 1:90, allowing the solution to be incubated at room temperature for 5 min. NBS oxidizes the thio-organophosphorus compounds by generating their respective oxidized analogs, which are more toxic for the enzymes. Percentage (%) of inhibition is reported for each enzyme with each toxicant. All the values are the means of measurements taken from three independent experiments, the error bars representing the standard deviations; (**c**) The POX dose-response plot, using K42R/K61R mutant electrochemical biosensor. Each value is the mean of measurements taken from five independent experiments, the error bars corresponding to the standard deviations d) Evaluation of the stability of the enzyme based assay during a period of 4 months. The biosensing MEPs were stored at 4 °C. I% measurements were performed using, 0.5 mM NaAc concentration and 5 s POX exposure time, for 1 µM POX. Each value is the mean of measurements taken from three independent measurements, the error bars corresponding to the standard deviations.

In preliminary experiments, the inhibition profile of the K42R/K61R, the WT and other EST2 mutants were evaluated (Fig. 2a,b). Details of the different mutants are reported in the SI. Each enzyme was immobilized on a different well of the electrochemical platform and the inhibition measurement performed as already described. The inhibition of the enzymes by each OP was converted to a percentage inhibition by comparing the response to a measurement taken in the absence of the OP.

Results are reported in Fig. 2a. The inhibition produced by each OP at a concentration of 1 μM with each enzyme demonstrates clear differences. Only K42R/K61R, WT and five other mutants (E50A, R31A, K61A, D145A, S36C, see SI) were completely inhibited by 1 μM POX (Fig. 2b), under the given conditions. Among these, K42R/K61R resulted to be more affected by a higher number of different OPs (with higher inhibition percentage) in comparison to the other mutants. This inhibition profile confirms the possibility to use this mutant for the biosensor development. Specifically, the mutant is active until the arrival of the OP that affects the electrochemical signal by covalent and stable binding to the crucial S155 in the active site (Fig. S1)^33,34^.

However, the inhibition profiles reported in Fig. 1a, due to the inhibition of each of the enzymes by each of the OPs at the same concentration could be useful to define inhibition patterns that allow the differentiation of the OPs. Indeed, the developing of a dedicated software for the deconvolution of the signals to identify each specific OPs by using the different mutants, will be explored in a future work.

As reported above, in the present study, the double mutant K42R/K61R was chosen as active part of the biosensor array. Experimental measurements were carried out in order to define the optimal enzyme concentration as well as substrate type, concentration, and incubation time. The results are reported in SI and in Fig. S3. Briefly, NaAc was chosen, among the other substrates, because it gives a reproducible electrochemical signal. Because POX determination involves an irreversible inhibition of the enzyme, the lowest feasible concentration of enzyme is necessary to reach a low detection limit. A value of 1 U/mL was experimentally chosen as optimal value. Inhibition measurements were carried out at optimized substrate concentration and time of 0.5 mM and 30 sec, respectively.

In Fig. 2c, it is reported the dose-inhibition curve (POX amount ranging between 1 to 10000 femtomoles) of the enzyme activity after exposure to POX, using K42R/K61R mutant. By means of the electrochemical measurement, it was possible to detect extremely small amount of the toxic compound in a short time (5 s of exposure time). A detection limit of 0.4 nM (0.4 femtomoles in 1 µL of POX solution) calculated as the concentration giving 10% inhibition^42^, was obtained by plotting the values of I % vs. inhibitor concentration and by using the equation (2):

2 $$y=4+\frac{105}{1+{(\frac{x}{43})}^{0.59}}$$

The concentration giving 50% inhibition (EC_50_) resulted to be 43 nM (corresponding to 12 mg/m^3^ of POX). Reproducibility was also assessed. Thus, five different bio-sensing MEPs were tested with the same concentration (100 nM) of POX resulting in a relative standard deviation percentage (R.S.D. %) value of 20 (inter-electrodes R.S.D.%). The biosensing MEP exceptional stability was checked weekly for 4 months, without loss of enzyme activity (Fig. 2d), when stored at 4 °C.

Immediately Dangerous To Life or Health (IDLH) Values for Parathion (thio-analog of POX) has been reported to be 10 mg/m^3^; the minimum lethal oral dose range from 0.17 to 1.471 mg/kg^43^; an oral dose ranging from 0.17 to 1.471 mg/kg is equivalent to a worker being exposed to about 8 to 69 mg/m^3^ for 30 minutes, assuming a breathing rate of 50 liters per minute and 100% absorption. Thus, considering these data, and elaborating the info evaluated from the calibration plot after 5 s exposure time, the developed biosensing platform posses enough sensitivity to be used as alarming system.

**Figure 3:**
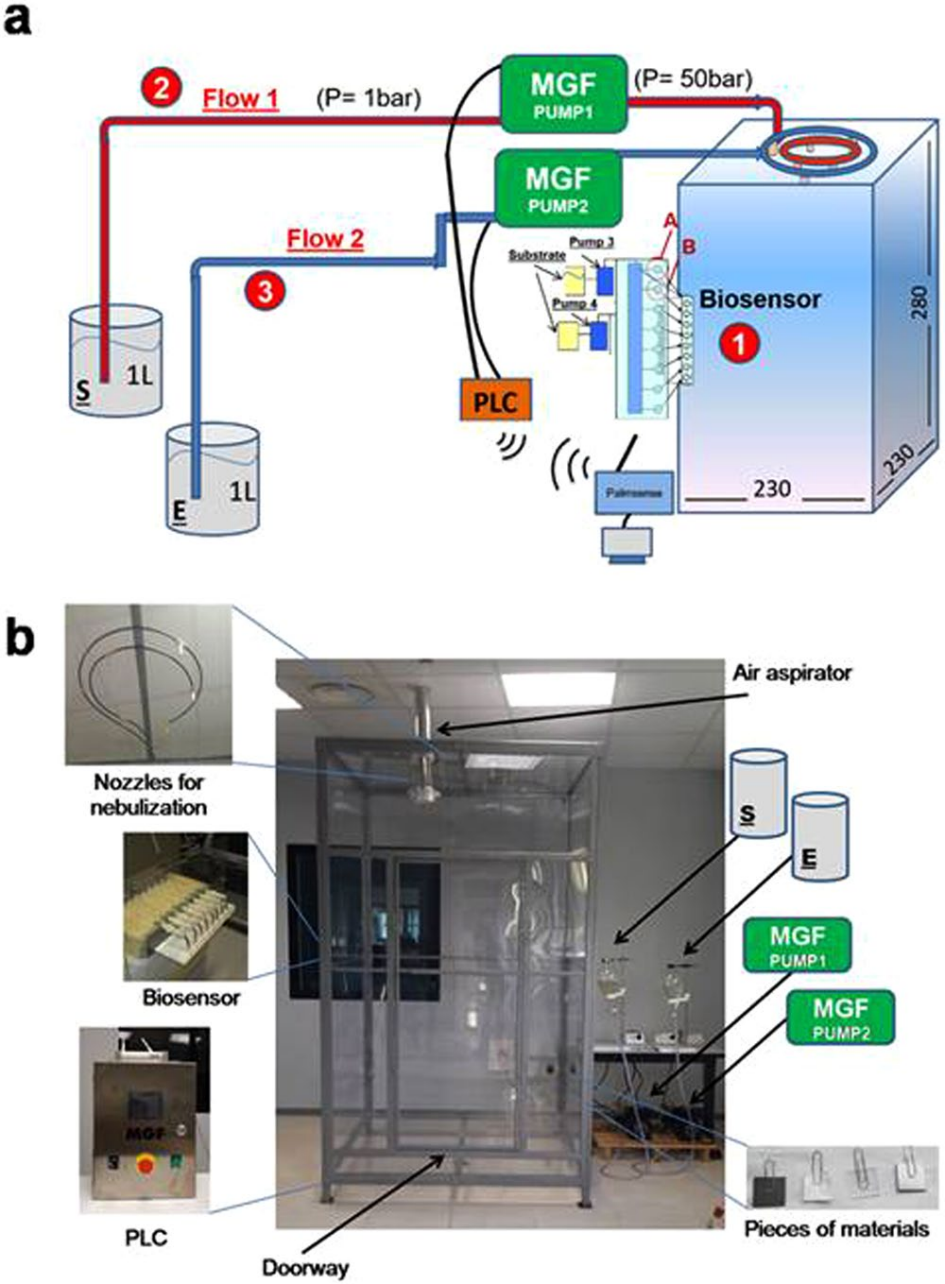
Demonstrator chamber. The inhibitory effect of POX on the EST2 biosensing platform was evaluated by nebulizing the NAS solution (100 µM POX, 100 µM MPOX, 25 µM MPTON), in the demonstrator chamber (2.30 × 2.30 × 2.80 m, Scheme (**a**) and Real view (**b**), by using 2 pumps (MGF1 and 2 for enzyme (E) and NAS (S) respectively) and a control system (PLC and see SI).

The assay set-up was tailored to the demonstrator shown later in Fig. 3 (scheme a and real view b) by developing the fluidic apparatus reported in Fig. 3b.

### Detoxification with free enzymes in water and on selected materials

The selected PLL enzymes (*Sso*Pox, *Sac*Pox, *Sso*W263F and *Sso*3Mut) when used alone at defined concentrations demonstrated high detoxification performances on POX spotted on a cotton strip, in the presence of detergents (SDS or commercial soap)^28^ and organic solvents (ethanol or methanol) (Fig. S2 and SI text). We also monitored each of the four enzymes on NAS substrate at two different concentrations (25 and 50 µg/mL) with or without SDS (0.025% w/v) (Table S2), showing that although *Sso*3Mut was the best, it was not able to destroy all of the NAS substrate (about 80% of efficiency).

Therefore in the next experiment, we managed to test the ENZYMIX4 with the NAS defined above and looking at its behavior with or without SDS (0.025% w/v). In particular, experiments of contamination/decontamination of buffered water solutions were setup by using enzymes at the concentration of 50 µg/mL each, except for *Sac*Pox, which was used at the concentration of 200 µg/mL. Under the selected conditions, detoxification of the NAS approached 100% as shown in Fig. S4, suggesting that *Sac*Pox complemented the S*so*3Mut specificity.

Having assessed the conditions for optimal degradation of NAS in water, contamination/decontamination tests were set up for different materials and type of surfaces, using the ENZYMIX4 formulation and the NAS. We did choose four materials namely glass, aluminum, cotton and linoleum based on the more common materials that can be found in a public setting and representing different absorbance capability.

Samples of these materials obtained from local suppliers, were cut in pieces (2.0 × 2.0 cm) and each piece contaminated with the NAS containing POX, MPOX and MPTON at 1.0, 1.0, and 0.25 mM concentration, respectively. The NAS (675 μl in total) was loaded onto each surface in 1–2 microliters aliquots. After complete absorption/desiccation of the solution (around 30 minutes for cotton and linoleum, 60 minutes for aluminum and glass), each contaminated piece was sunken into the ENZYMIX4 solution (3 ml of 20 mM Hepes pH 8.5, 4% acetonitrile). After 60 min incubation at 21 ± 2 °C, pieces were removed and the amount of *p*-NP in the solution read as usual. This allowed evaluating the efficiency on NAS (Table S3). As shown in the Table for cotton, aluminum, and glass, 85.6, 84.5 and 76.0% hydrolyses were obtained respectively after 60 minutes incubation. In the presence of 0.025% (w/v) SDS for cotton and aluminum there was an improvement in degradation (90.8 and 89.2% respectively). Only for linoleum, the detoxification process was less efficient (28.9% degradation). Likely, the porosity and/or the adsorbing properties of the linoleum surface trap in some way the pesticides making them not available to the hydrolytic action of enzymes. The studies of the theoretical behavior of NAS on absorbing surfaces is still in its infancy as are the experimental validations of such theories with respect the detoxification/decontamination means^44^. This will likely be an area of further investigation in the future. Carboxylesterases could also be used at the end of the decontamination process to completely block any residual agent escaped to the hydrolysis according to the principle that “clean is clean”. In further studies we could also think to the use of carboxylesterases to destroy carbamates and pyrethroids with the assistance of oximes^22,45^.

In conclusion, these experiments provide a proof-of-principle of the possibility to develop enzyme formulations containing combination of stable enzymes for the efficient destruction of mixtures of organophosphates in aqueous solutions and on different surface’s type and materials.

### Detoxification with nebulized enzymes

To test a prototype of a decontamination mixture useful for the treatment of confined spaces and contaminated materials contained in it, a bench-size simulator (Fig. S5) has been built. The simulator is made of a box (150 × 40 × 40 cm) in polycarbonate (Lexican) equipped with an airtight sealing removable cup, a pump system with flux regulators, two nebulizers nozzles (100 μm) on one side and an air filter on the other. This filter is made of activated charcoal through which the air can been aspired at the end of the experiment and forced to bubble into a solution of concentrated (5 M) NaOH, as a trap for residual OPs.

We worked out all the preliminary (small-scale) experiments of OP degradation on liquid, tissues, surfaces and airstream in this box. We performed two kind of experiments. In a first set of experiments, a solution (100 μM) of the nerve agent simulant POX was placed inside the box in an open plate or was spotted on pieces of the different materials cited above. After that, single enzymes (Table S4 and text) or the ENZYMIX3 solutions were nebulized over the plate and the surfaces of different materials and left to stand up to 20 minutes before analysis. In a second set of experiments, POX was nebulized from a second pump and nozzle, together with the enzyme(s) and the condensed mist recovered through a funnel communicating with the outside through a hole in the floor of the box and immediately read as above. Given the relevant amount of enzyme required to make meaningful tests, preliminary experiments in plates and on the mist were made only with POX and two *Sso*Pox mutants, separately.

At the end of each experiment the box was opened, the materials removed and samples (liquid in the plates and surfaces mimics) analyzed for the residual POX. After condensation of the mist, we monitored the treatment time and set out conditions for the catalytic action to occur completely. Reading the absorbance at 405 nm allowed quantitating the degradation in the plates, whereas extraction with acetonitrile and quantitation by Gas chromatography spectrometry (GC) was used for residual POX on the different materials. We calculated that by nebulizing 100 ml of 480 μg/ml *Sso*3Mut the amount of enzyme that falls on a plate containing 20 ml of 100 μM POX was enough to hydrolyze 87.5% of the substrate in 20 min. Therefore, for the experiments in the airstream we used less enzyme (ranging from 100 to 510 μg/ml). *Sso*3Mut was nebulized together with POX, the condensed mist was recovered immediately through the funnel and *p*-NP was read to see the effect in the “air”. After 2 minutes from the starting of nebulization it was assessed that 44% degradation had occurred with 100 μg/ml enzyme. We also looked at the effect of ENZYMIX3 (wt *Sso*Pox was excluded to reduce cost of the experiment; 200 mg *Sac*Pox, 50 mg *Sso*3mut, 50 mg W263F) on the 2.0 × 2.0 cm of the four materials mentioned above placed inside the box and recovered after 20 min from the starting of the nebulization. The residual POX was extracted with 4 ml of acetonitrile and quantitate by GC. It was observed that less than 1 nanomole of POX was present per cm^2^.

### Kinetics of used enzymes with “live” nerve agents

To further support the use of *Sso*3Mut as bioscavenger and assess the effect of the introduced mutations we analyzed its activity against “live” nerve agents of the “G serie” cyclosarin, sarin, soman, tabun, and VX, at 37 °C, and compared it with the other enzymes. The incubation of the enzyme with each nerve agent and the recording of AChE inhibition at different times allowed us to calculate the detoxification kinetics (Table 1)^46^. The mutant *Sso*3Mut had remarkable activity, but a negligible effect on VX. Notably, the obtained values are 10^2^ away from those calculated for the best mutant of *Pseudomonas diminuta* PTE (H257Y/L303T), obtained by a combinatorial strategy of rational design and directed evolution that show hydrolytic activities of 2 × 10^6^, 5 × 10^5^, and 8 × 10^5^ M^−1^ s^−1^ toward sarin, soman and cyclosarin, respectively^47^. It has also to be stressed that by using POX as the simulant of nerve agent(s) in our *in vitro* evolution approach the discovery process of new efficient variants is completely blind; nonetheless POX seems to act as a good surrogate to keep on in evolving new variants. Finally, even though PLL variants seem less efficient with respect to mesophilic PTE, it has to be stressed that thermostability provides unrivaled advantages in terms of easier enzyme purification, longer shelf life and stability under many different stressing conditions.

**Table 1:** Kinetic analyses of thermostable PLLs including *Sso*Pox, its two variants W263F and *Sso*3Mut and *Sac*Pox towards hydrolysis of “live” nerve agents.

	*k*_cat_ (min^−1^)		*k*_cat_/K_M_ (M^−1^s^−1^)	
	*Sac*Pox *wt*	*Sso*Pox *wt*	*Sso*W263F	*Sso*3Mut
Tabun	n.a.	n.a.	n.a.	4.12 ± 1.04 × 10^3^
Sarin	n.a.	n.a.	n.a.	1.66 ± 0.31 × 10^4^
Soman	n.a.	n.a.	n.a.	2.59 ± 0.67 × 10^3^
Cyclosarin	n.a.	n.a.	n.a.	4.58 ± 0.05 × 10^3^
(+)-Cyclosarin	0.0292 ± 0.0014^a^	0.1042 ± 0.0025^a^	0.0297 ± 0.0009^a^	n.a.
(−)-Cyclosarin	0.0218 ± 0.0011^a^	0.0309 ± 0.0012^a^	0.132 ± 0.0039^a^	n.a.
VX	n.a.	n.a.	n.a.	n.d.

^a^Data taken from Merone *et al*.^27^. n.a.: not analyzed; n.d.: not detected. Values are means ± the SD of measurements taken from three experiments.

### Assembling and testing an integrated system in a large chamber

For the realization of the large scale experiments described here there was the need to optimize the expression and the downstream treatment of biomass to reach the production level of grams amount of enzymes. The large-scale production of *Sulfolobus PLLs* has been recently reported^47,48^. Furthermore, large-scale production is a prerequisite for any real application in the field. To this aim, we used the optimized procedures for the large scale preparation of three enzymes. As reported in SI and Table S6 the amounts in grams obtained for *Sso*Pox, *Sac*Pox and *Sso*3Mut were 12.7 ± 0.2, 8.9 ± 0.2 and 15.1 ± 0.3 g, respectively.

We tested the biosensor and enzyme formulations in the large chamber of Fig. 3. The technical characteristics of the chamber are shown in scheme **a** and SI. The chamber (10 m^3^) can contain 4/5 people and is used to simulate an unsafe environment due to the chemical contamination. The new setting is made of the biosensor placed at a single position halfway on a wall as to detect the OP(s), almost immediately after nebulization (see SI). The four samples of materials to be detoxified (Fig. 3) were placed on the walls not containing the door, at three different heights (high, medium and low). The system of pumps that nebulise the simulant and the detoxification solutions together with valves, air aspirator, and the microfluidics dedicated to the biosensor were all operated remotely from a palmar PC. Specifically, the system was controlled by a programmable logic controller (PLC) and by two electro pumps; this made it possible to switch very quickly between the mist containing the nerve agent simulant and the mist of the detoxification solution.

For each experiment 1 L of Nerve Agent simulants (NAS) (100 μM POX, 100 μM MPOX, 25 μM MPTON) and 1 L of ENZYMIX3 (2 g of *Sac*Pox, 0.5 g of *Sso*W263F and 0.5 g of *Sso*3Mut) were used. As a control the effect on the biosensor of the airstream in the absence of the NAS was evaluated.

Thus the biosensor was exposed to 1 μL of deionized water in the ambient airstream of the demonstration chamber for 5 s. Then, the response of the biosensor (I_0_) was monitored, by dispensing the substrate solution (0.5 mM) in the well for 10 s. Afterwards, the flow was switched off and the substrate incubation lasted for 20 s before starting the DPV measurement. To measure the I_x_ the same procedure was adopted but, in this case, the biosensor was exposed to the NAS mist stream for 5 s. After 5 s of mist flowing in the chamber, the substrate solution was dispensed in the well of the MEP (20 s) and then, the measurement was performed. This time was sufficient to obtain a 100% degree of inhibition.

It is important to note that the mist flowed nearly perpendicular over the surface of each biosensor well. Indeed, the biosensing platform was fixed on a lateral wall, below the entrance of the gas stream in the demonstrator chamber.

A home-made script allowed to monitor the current measured by the biosensing platform and to activate the electro pumps.

The script commanded to switch on the pump via wireless when the measured current (I_x_) is around 30% of the I_0_ current value (eq. 1), allowing the detoxification solution entering in the demonstrator chamber.

This demonstrated that the procedure of exposing the biosensing platform to the nerve agent simulant flow is an effective approach for setting an alarming system.

The complete protocol of the experiment comprising the biosensor activation and working up is reported in the SI and Methods. In our experiments, as said above we also tested the effect on the same materials tested in the small box. As for the small box we performed preliminary experiments with single enzymes and single substrates.

The results with the NAS were even better than the ones reported above for the small box. The residual amount of each neurotoxin was measured in two independent experiments (Table 2). In Exp.1 less than 10 picomoles/cm^2^ of MPOX were found on each surface, partly confirmed in Exp. 2 except that for Linoleum (residual amount of MPOX ranging from 230 to 195 picomoles/cm^2^). For MPTON and POX less efficiency was observed only on cotton in Exp. 2 (from 200 to 800 picomoles/cm^2^).

**Table 2:** Residual concentration of NAS extracted from the four surfaces placed on the three walls of the box after nebulization of ENZYMIX3.

Samples	MPOX (μmol/cm^2^)		MPTON (μmol/cm^2^)		POX (μmol/cm^2^)	
	*Exp*. *1*	*Exp*. *2*	*Exp*. *1*	*Exp*. *2*	*Exp*. *1*	*Exp*. *2*
Glass 1	<1*10^−5^	nd	2.6*10^−5^	1.5*10^−6^	<1*10^−5^	6.0*10^−5^
Glass 2	<1*10^−5^	nd	<1*10^−5^	nd	<1*10^−5^	4.95*10^−4^
Glass 3	<1*10^−5^	nd	<1*10^−5^	nd	<1*10^−5^	9.86*10^−4^
Aluminum 1	<1*10^−5^	nd	<1*10^−5^	4.35*10^−5^	<1*10^−5^	5.785*10^−4^
Aluminum 2	<1*10^−5^	nd	<1*10^−5^	1.15*10^−5^	<1*10^−5^	3.54*10^−4^
Aluminum 3	<1*10^−5^	nd	<1*10^−5^	7.5*10^−6^	<1*10^−5^	4.485*10^−4^
Linoleum 1	<1*10^−5^	2.23*10^−4^	2.0*10^−5^	2.5*10^−4^	<1*10^−5^	5.69*10^−4^
Linoleum 2	<1*10^−5^	1.95*10^−4^	<1*10^−5^	9.0*10^−6^	<1*10^−5^	1.00*10^−3^
Linoleum 3	<1*10^−5^	2.28*10^−4^	2.6*10^−5^	7.7*10^−5^	<1*10^−5^	7.57*10^−4^
Cotton 1	<1*10^−5^	nd	2.8*10^−5^	2.365*10^−4^	<1*10^−5^	5.09*10^−3^
Cotton 2	<1*10^−5^	nd	<1*10^−5^	2.1*10^−4^	<1*10^−5^	7.965*10^−3^
Cotton 3	<1*10^−5^	nd	1.8*10^−5^	8.35*10^−4^	<1*10^−5^	2.08*10^−3^

Assays were in triplicate.

In the same experiments, we also collected every 30 s samples of condensed mist at times ranging from 3 to 12 minutes and analyzed by GC the residual content of each compound. For MPOX there was an amazing 1.5*10^5^ folds reduction in the concentration soon after 3 minutes from the starting of nebulization (Table 3). The concentration raised 10-fold more at 4.0 minutes but starting from 4.30 minutes MPOX was no more detectable. For MPTON the concentration was 10^−4^ μM (2.5*10^6^ fold less than the starting concentration) up to 3.5 minutes and then decreased steadily to become undetectable at 12 minutes. The behavior with POX was similar to MPOX.

**Table 3:** Residual amount of NAS extracted from collected condensed samples (mist). Starting concentrations were 100 μmol/L.

Samples (min)	MPOX (μmol/L)	MPTON (μmol/L)	POX (μmol/L)
1 (3.00)	6.76 ± 1.25*10^−4^	0.97 ± 0.075*10^−4^	8.41 ± 1.15*10^−4^
2 (3.30)	4.79 ± 0.6*10^−4^	1.00 ± 0.05*10^−4^	8.49 ± 1.15*10^−3^
3 (4.00)	1.40 ± 0.12*10^−3^	2.40 ± 0.16*10^−5^	1.37 ± 0.10*10^−3^
4 (4.30)	nd	6.75 ± 1.15*10^−5^	nd
5 (6.30)	nd	5.30 ± 0.75*10^−5^	nd
6 (12.00)	nd	nd	nd

Assays were in triplicate and reported data are the means of two independent experiments within the indicated ranges.

Nd = not detectable.

## Conclusions

The high toxicity to human of OP nerve agents has led in last decades to research of effective antidotes. Since the standard treatments have limited efficacy in case of nerve gas poisoning and are inadequate to prevent incapacitation^22^, a promising alternative approach is the development of catalytic bioscavengers^23^. Human enzymes, such as the paraoxonase enzymes, are the better choice for this kind of application^23,46^, but microbial and stable enzymes are more attractive for skin, air and surface decontaminations^49^. Our careful studies of generation, preparation and characterization of formulations of thermostable PTE-like enzymes have demonstrated for the first time, to the best of our knowledge, the possibility to use thermostable enzymes in an integrated system to detect and quickly detoxify nerve agents simulants on different surfaces of different materials and most importantly starting from the air. The strategy of “detection and reaction” based on the prototypes described here could be of deterrence for terroristic acts based on deadly nerve agents on which our enzymes have been demonstrated to be surprisingly active with respect to *Pseudomonas* PTE, also considering that measurements were performed at room temperature. Nerve-agent hydrolyzing efficiencies of the *Sso*Pox3Mut of about 2 × 10^4^ M^−1^ s^−1^ could be relevant for CWNA decontamination meaning that 1 μM GB could be degraded in a few minutes with 1 nM of enzyme. Because we used OP pesticides as model compounds it also emerges from this study that this apparatus appears to be ideally suited to detect and identify low concentrations of pesticides in the environment (due to the run-off from cotton farms, for example).

## Materials and Methods

### Chemicals

All organophosphate pesticides used for kinetics or as markers were purchased from Sigma Chemical Co. (St. Louis, MO). The Bundeswehr Institute of Pharmacology and Toxicology (Munich, Germany) provided cyclosarin (GF), tabun (GA), sarin (GB) and VX. Molecular mass markers for SDS-PAGE were obtained from Bio Rad (Hercules, CA). Kartell Labware provided the commercial soap (LM1).

All the media components for *E*. *coli* growth were from Sigma-Aldrich (Italy) except for the yeast extract (Organo-Technie, France) and the tryptone (OXOID, UK).

### Methods

#### Cloning

Most of clones used here were described in other works^27,28,31,33,40,41^, except for the mutational analysis of EST2 K42 and K61. Starting from the pT7-7-EST2 construct, we mutagenize the residues in R, as single and double mutant. For these site-direct mutagenesis experiments we used the QuikChange Lightning Site-Directed Mutagenesis Kit (Agilent Technologies, CA, USA), following the manufacturer’s instructions, and using as mutagenic primers complementary pairs of oligonucleotides: EST2 K42R forward 5′-CCTGTCAAGCGCGAGCCCGTGGCCGAG-3′; EST2 K42R reverse 5′-CACGGGCTCGCGCTTGACAGGAGGAAAC-3′; EST2 K61R forward 5′-CGCACGCTCCGCGTGCGCCATGTACCGCC-3′; EST2 K61R reverse 5′-CATGCGCACGCGGAGCGTGCGGCCAGG-3′. After mutagenesis the new mutants were controlled by DNA sequencing analysis to exclude the presence of undesired mutations.

#### Selection of enzymes

EST2 was choosen as biosensor platform because being the most studied of our carboxylesterases showed the best characteristics in terms of high activity, sensitivity to many OPs, stability, good expression in *E*.*coli* and ease purification, availability of several mutants^31,33,35^.

*Sulfolobus* PLLs and single variants in part already analysed in other works^27,28,40^ and in part analysed here were mixed and probed against POX or NAS. Preliminary tests were made in buffered water solutions and in the small box with single enzymes or mixtures. We tested different concentrations (Table S2) and took into account the behaviour under different stress conditions (Fig. S2) as well as the specificity shown in Table S1. From these data we decided the enzymes and their concentration ratios in the mixtures. Assays requiring reading of *p*NP were made spectrophotometrically or by HPLC whereas pesticides were measured by HPLC or GC-FTD.

#### Preparation of enzymes

EST2 and variants were purified on small scale (mg amounts) essentially as described in Pezzullo *et al*.^35^. Small-scale production of PLLs was as described^41^.

The enzymes *Sac*Pox, *Sso*Pox, *Sso*W263F and *Sso*3Mut were produced on large scale starting from the engineered strains described above by high cell density fermentations (see also SI)^47,48^.

The enzymes were extracted from the fermentation final biomasses (60 Liters from a 150 L fermentor) by using a high-pressure cell homogenizer (Emulsiflex C3, Avestin, Germany). A pressure of 15000-20000 psi was used to break the wet biomass (1 Kg) at once, re-suspending the cells in an extraction buffer (20 mM Hepes, 0.2 mM CoCl_2_ or 0.2 mM MnCl_2_, 0.1% (w/v) Triton X, pH = 8.5), in a ratio of 1 to 3 (w/v). Thermo-precipitation of the crude extracts was performed at 70 °C for 25 minutes and at 500 rpm in a 25-L glass jacked bio-reactor (Steroglass, Italy) at a protein concentration of 4 g∙L^−1^. The thermo-precipitated solutions were further purified in two steps by ultra-filtration on membranes using an automatic tangential flow filtration system (TFF Uniflux 10 system, GE Healthcare, USA)^50,51^. The first step was performed on 100 kDa cut-off membranes. The permeated volumes, which contained the enzymes, were then concentrated on 5 kDa membranes. The concentrated solutions were freeze-dried (Epsilon 2–6 D, Christ, Germany). When required, a further purification step was performed by anionic exchange on a Q-Sepharose FF column (2.6 × 10 cm; GE Healthcare, USA).

Enzymes were kept separated in lyophilized form until mixing a few days before the experiment and the mixture was perfectly stable for at least one week.

#### Electrochemical apparatus and measurements

The Multichannel electrochemical platform (MEP) was based on 8 screen-printed electrochemical cells, deposited side by side on a plastic substrate, consisting of a carbon WE (diameter ≈ 2 mm), a silver RE, and a graphite CE^52^ (Fig. 1).

The biosensing platform was developed by modifying the surface of each of the 8 WE of the array (Fig. 1) by drop casting 2 µL of a solution containing 1 U/mL of enzyme, 1% CMC and 1 mg/mL BSA in 20 mM phosphate buffer (pH 7) containing 100 mM KCl (PB). After the modification of each WE, a 8-well methacrylate box (4 mm × 84 mm × 5 mm) is fixed onto the platform by using a double layer adhesive. Each well is 8 mm in diameter and it is positioned exactly in correspondence of each electrochemical cell of the array. The biosensing platform is stored a 4 °C till use.

The electrochemical measurements were carried out with an automated flow-based system. The flow system was comprised by peristaltic pumps (Gilson) coupled to a valve system controlled by a PLC, a custom flow-nozzle system, and a commercial portable and miniaturized potentiostat (Palmsens, Netherlands) (Fig. 3a). The potentiostat was custom modified in order to receive input and to send data from and to the PLC. The reagents (0.5 mM 2-naphthyacetate (NaAc) in 20 mM phosphate buffer, pH 7.0 containing 100 mM KCl, deionized water) were arranged in different reservoirs and the measurements were performed at 25 °C. The substrate flow, generated by the peristaltic pumps, delivered the solution to each electrochemical cell of the array, through the nozzle system, with each nozzle positioned directly on the WE (Fig. 3b). With this type of set-up, it was possible to reach each well selectively and consecutively with the reagents by means of valve control. Each well is filled in less than 10 s. Then the flow is switched off and the substrate incubation lasted for 20 s before starting the electrochemical measurement. Differential Pulse Voltammetry (DPV) was used to monitor the electroanalytical signal due to oxidation of 2-naphthol generated by enzymatic hydrolysis of NA (potential range 0–+ 0.9 V, step potential 0.005 V, amplitude 0.07 V, scan rate 0.033 V/sec). All potentials were referred to the Ag/AgCl pseudo-reference electrode. Each well (and the corresponding electrochemical sensor) was used for a single measurement. Indeed, is known in literature that phenol-derivatives can cause electrode fouling. Thus, using the sensor as disposable, this phenomenon can be avoided. The current measurement was performed always in the presence of 0.5 mM NaAc in 20 mM phosphate buffer, pH 7.0 containing 100 mM KCl.

#### Dose response curve and optimization experiments

The inhibition rate was calculated as I% = [(I_0_ − I_x_)/I_0_ * 100] where I_0_ is the current intensity recorded for the measurement in the absence of inhibitor and I_x_ is the current recorded for measurement in the presence of inhibitor^42,53–55^.

Generally, the I_0_ measurement was performed at the beginning of each working day. I% was referred to this measurement. However, the I_0_ measurement was repeated time to time in order to check full activity of the enzyme during the working day.

I_0_ measurements were performed by depositing on the surface of the WE, 1 µL of deionized water. Deionized water was also used to prepare the solution of the POX standards and simulant spray. After 5 s, 100 µL of the solution containing the substrate (0.5 mM NaAc in PB) was added in the well using the peristaltic pump. After 20 s, the electrochemical measurement was performed and automatically saved by the software.

For the dose-response plot and all the optimization experiments, 1 μL of toxicants was dispensed on a new well, and after 5 sec the enzymatic substrate was dispensed, following the same procedure reported above. Each different sample (i.e. different concentration of the same OP or different OPs) were tested in the different wells of the array. All of the toxicants, with the exception of POX were diluted in PB containing N-bromosuccinimide (NBS) in a ratio of 1:90, allowing the solution to be incubated at room temperature for 5 min. NBS oxidizes the thio-organophosphorus compounds by generating their respective oxidized analogs, which are more toxic for the enzymes.

#### Analyses in the demonstrator chamber

In the demonstrator chamber, the measurements were performed using a toxicant mist (simulant spray) for the inhibition test.

The I_0_ measurements were performed by exposing the biosensor just to 1 μL of deionized water for 5 s. Then the PLC activates the peristaltic pumps, for 10 s to dispense the substrate solution. After 20 s, the electrochemical measurement was performed and automatically saved by the software. Each measurement was repeated in a new well.

The inhibition measurements (I_x_) were performed by exposing the MEP to the simulant spray. For this measurement a new well was used. After 5 s, the substrate solution was dispensed. Each operation was controlled by the PLC. A home-made script allowed to monitor the current measured by the biosensing platform and to activate the electro pumps. When the measured current (I_x_) is around 30% of the I_0_ current value one pump is switched on via wireless and the detoxification solution enters in the demonstrator chamber.

#### Esterase activity

*p*NP-hexanoate hydrolysis was followed by monitoring of *p*-nitrophenol production at 405 nm, in 1-cm path-length cells with a Cary 100 spectrophotometer (Varian, Australia) as previously described^31^. Briefly, assays were performed at 70 °C the optimal temperature of the wild type enzyme and mutants, in a mixture of phosphate buffer (40 mM; pH = 7.1) containing acetonitrile (4%) and *p*NP-hexanoate (100 μM) (standard assay). Stock solutions of *p*NP-hexanoate, were prepared by dissolving substrate in pure acetonitrile. Samples of identical composition as the assay mixture, omitting the enzyme, provided suitable blanks. Assays were done in duplicate and results were means of two independent experiments. One unit of esterase activity was defined as the amount of the protein releasing 1 µM of *p*-nitrophenoxide/min from *p*NP-hexanoate at indicated temperature. The absorption coefficient used for *p*-nitrophenoxide was 21,000 M^−1^ cm^−1^ (at 70 °C and pH = 7.1). Kinetic parameters on *p*NP-hexanoate were measured by ranging the substrate from 0.025 to 0.2 mM. Initial velocities versus substrate concentration data were analysed with the GRAFIT program (Grafit Version 3.0, Erithacus Software Ltd., UK).

#### Hydrolysis of OP pesticides

Hydrolysis of OP pesticides in buffered water and in the presence of solvents and detergents was followed spectrophotometrically as described^28^. Procedures of contamination and decontamination of materials were as reported^28^.

#### HPLC and GC-FTD analytical methods

For the POX analyses immediately after sampling (from the mist or different materials) acetonitrile was added in order to deactivate the enzyme(s) and extract the compounds. The samples were concentrated (1 ml final volume) under nitrogen flow and aliquots injected onto a C18 Discovery phase column (25 cm ×4.6 mm ×5 micron) run on an HPLC apparatus (Shimadzu 20 series Class) according to published procedures^56^. An isocratic elution step in acetonitrile (90%) followed. Absorption reading (XRF 10 fluorescence UV-Vis detector 10 series) was at λ = 275 nm. External calibration curve for paraoxon is reported in Fig. S6a. For HPLC analysis of *p*-NP^57^ the sample was acidified (by adding an appropriate volume of a 1% solution of sulfuric acid to have a 0.1% final concentration). Upon acidification the absorption peak of *p*-NP moves from 405 nm to 317 nm. Runs were made on a C18 Discovery phase column (25 cm × 4.6 mm × 5 micron). An isocratic elution step in acetonitrile (90%) followed.

The mixture of pesticides (POX, MPTON and MPOX) was analysed by a Gas chromatographic (GC) analysis method^58,59^. A Gas chromatograph Shimadzu GC 2010 series- equipped with a Flame Thermionic Detector (FTD) was used. A 30 mt Rtx-5 Restek column (0.25 mm ID), was run under the following conditions: Temperature oven ramps up to 40 °C; hold for 1 min then up to 100 °C at a rate of 40 °C/min; hold for 1 min then up to 200 °C rate 40 °C/min; hold for 8 min. Injections were done in split of 1 :10 at 250 °C. Standards used were MPOX (Pestanal), MPTON (Pestanal) and POX (Pestanal). External calibration curves are reported in Fig. S6b–d. Standards or unknown samples were dried under nitrogen and re-suspended in pesticide-free hexane before injection. Retention times for MPOX, MPTON and POX were 13.419, 14.207 and 14.533 minutes respectively.

#### Hydrolysis of GA, GB, GF and GD by *Sso*3Mut

*Sso*3Mut was incubated with 500 µM GA, 80 µM GB, 100 µM GF or 60 µM GD in Tris-HCl buffer (0.1 M, pH = 7.4, 37 °C). Aliquots were taken up to 60 minutes (GA up to 90 minutes) and transferred to a phosphate buffer solution (0.1 M, pH = 7.4), containing DTNB and acetylthiocholine (0.3 and 0.5 mM respectively). The reaction was started by adding human AChE. The absorbance was followed for 5 minutes and the inhibition curves were studied by non-linear regression analysis to get the first order inhibition rate constant *k*_1_, which was then plotted versus time to get the detoxification constant *k*_obs_. Data were corrected for spontaneous OP hydrolysis and finally *k*_cat_/K_M_ value was calculated (n = 3) as described in ref.^22^.

#### Building up the small box and the demonstration chamber

The small box (Fig. S5) and the demonstration chamber (Fig. 2b) were built of the material Lexan, a polycarbonate synthetic resin tough and transparent. The small box was realized from a local manufacturer following our graphical designs and was kept in the lab having bench-size dimensions. The large demonstrator was realized by workers of the Tecno Bios company (Bn, Italy), again following graphical designs and our instructions. The demonstrator was located in a large room and equipped with an air aspirator as shown in Fig. 3b. The biosensor was located on the left wall of the demonstrator chamber. Pump 3 and pump 4 are peristaltic pumps used to add the substrate for the biosensor in the channel A and B, respectively; Pumps MGF1 and MGF2 are electropumps for nebulization of pesticides and enzymatic solution in the room, respectively. All the devices were automatically controlled by a programmable logic controller (PLC).

#### Workflow of automated steps for nebulization experiments controlled by PLC

The PLC has been set up to automatically control the action for the simulation of contamination/decontamination experiments with 19 Steps at specified times (min: sec). 1_(00:00): START Pump 3; 2_(00:15): STOP Pump 3; 3_ wait; 4_(01:00): START MGF Pump1; 5_ wait; 6_ (01:32): START Pump 4; 7_(01:47): STOP Pump 4; 8_wait; 9_(02:32): START Pump MGF2: 10_(03:00): Collect Sample 1; 11_(03:30): Collect Sample 2; 12_(03:31): STOP Pump MGF1; 13_(04:00): Collect Sample 3; 14_(04:20): STOP Pump MGF2; 15_(04:30): Collect Sample 4; 16_(06:20): START Air aspirator; 17_(06:30): Collect Sample 5; 18_(12:00): Collect Sample 6; 19_(30:00): STOP Air aspirator. The time between step 8 and 9 was under the control of the positive response from the biosensor.

### Statistic analysis

Since in many cases the number of experiments are limited (<5) generally we reported the mean values and the corresponding ranges; otherwise, it is properly specified.

## Electronic supplementary material

Supplementary Information
