# Supplementary material for: Innovative Biocatalysts as Tools to Detect and Inactivate Nerve Agents
Source: Sci Rep. 2018 Sep 13;8:13773. doi: 10.1038/s41598-018-31751-5 (PMC6137069; doi:10.1038/s41598-018-31751-5)
Supplement: Supplementary file 1 — Supplementary Information [file 41598_2018_31751_MOESM1_ESM.docx]

**Supplementary Information**

**Innovative Biocatalysts as tools to detect and inactivate nerve agents**

**Elena Porzio^1^*, Francesca Bettazzi^2^*, Luigi Mandrich^1^, Immacolata Del Giudice^1^, Odile F. Restaino^3^, Serena Laschi^4^, Ferdinando Febbraio^1^, Valentina De Luca^1^, Maria G. Borzacchiello^3^, Teresa M. Carusone^1^, Franz Worek^4^, Antonio Pisanti^5^, Piero Porcaro^5^, Chiara Schiraldi^3^, Mario De Rosa^3^, Ilaria Palchetti^2^, Giuseppe Manco^1#^.**

^1^ E. Porzio, L. Mandrich, I. Del Giudice, F. Febbraio, V. De Luca, T. M. Carusone, G. Manco,

Institute of Protein Biochemistry, National Research Council of Italy, Naples, Italy.

^2^ F. Bettazzi, I. Palchetti, Department of Chemistry, University of Florence, Sesto Fiorentino (FI), Italy.

^3^ O. F. Restaino, C. Schiraldi, M. De Rosa, University of Campania "Luigi Vanvitelli", Naples, Italy.

^4^ S. Laschi, Ecobioservices and Research srl, Modica (RG).

^5^ F. Worek, Bundeswehr Institute of Pharmacology and Toxicology, Munich, Germany.

^6^ A. Pisanti, P. Porcaro, Tecno Bios srl, Italy.

**^#^**correspondence to: g.manco@ibp.cnr.it,Institute of Protein Biochemistry, National Research Council of Italy, Via P. Castellino 111, 80131, Naples, Italy

**Selection of the best EST2 mutant for a biosensor**

In preliminary experiments we exploited wild type EST2 and successively a variant selected from a small library of mutants produced in another study,^[1]^ as the active part of the biosensor; the latter variant (K42R/K61R) was selected because found in the present study to be more efficient and more sensitive to OPs with respect to the wild type. Shortly, focus was on mutations at residues K42 and K61 (positions shown in the EST2 structure Fig. S1a), which were found to increase enzyme kinetic thermal stability when singularly or both mutated into R. In particular, the mutants K61R and K42R displayed half-lives of 690 and 540 minutes at 70 °C, around 3- and 2.25-fold higher with respect to the wild type respectively (Fig. S1b). The double mutant instead was 1.4 fold more stable with respect to the wt. The increased stability was observed also as overall fold stability (Tm values; Fig. S1c). In the case of single mutants were was a reduction of 5 and 0.5 degrees centigrade in K42R and K61R respectively, whereas the double mutant again appeared more stable than wt (1 degree C). This effect is not unusal when doing protein engineering, e.g. the effect of mutations is not always additive.^[2]^ In contrast with what is generally observed, namely a trade-off between activity and stability,^[3]^ here we observed a relevant raise also in the catalytic activity, registering for the double mutant K42R/K61R a value of 10,500 U/mg with respect to 7,500 U/mg observed for the wild type enzyme, using *p*-NP-hexanoate as substrate. The substrate specificity (*k*_cat_/K_M_) was substantially unchanged because the R mutants imparted only a slight increase of affinity (K_M_) value from 16 to 26 μM. The stability was comparable with that of wt EST2 (Fig. S1b and c).The mutant was purified as described.^[1]^


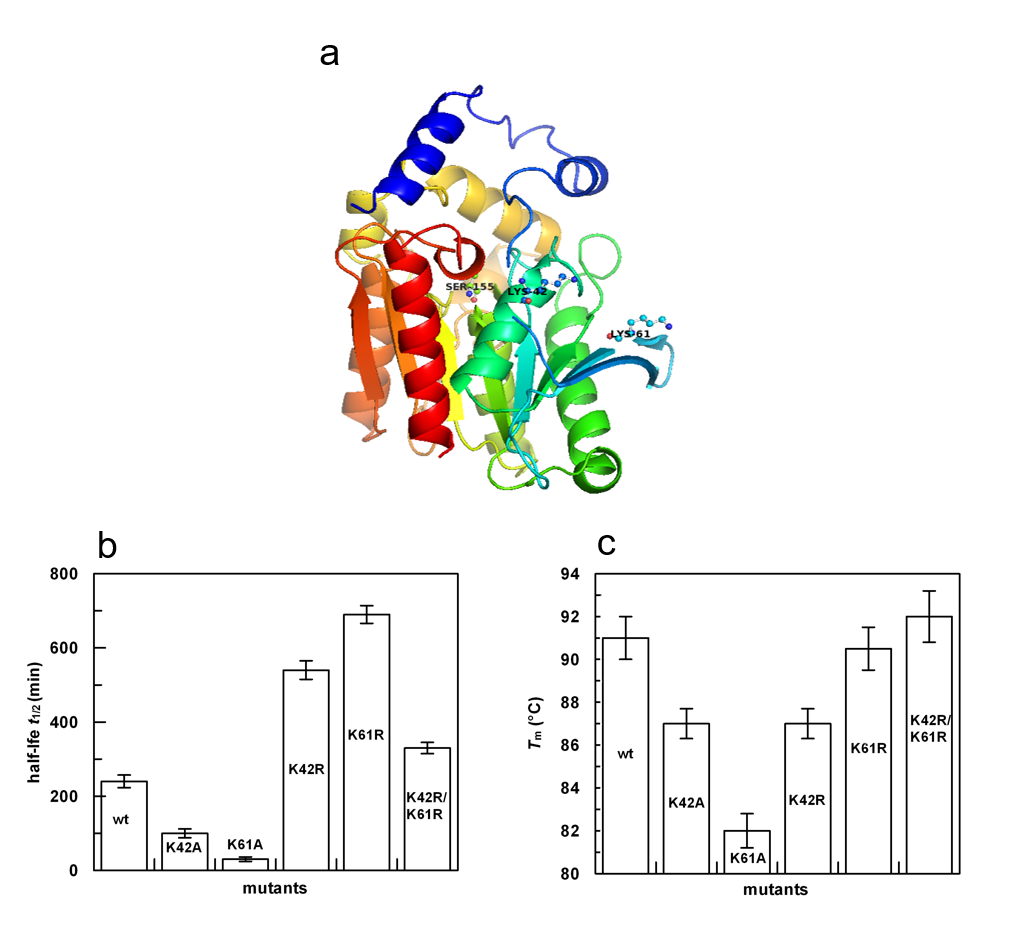


Figure S1 **a**) EST2 structure with highlighted S155, K42 and K61. Thermodynamic parameters of EST2 mutants. **b**) Histogram of the catalytic half-lives (t1/2) measured for EST2 and mutants as reported in Materials and Methods. **c**) Histogram of the melting temperatures (Tm) measured for EST2 and mutants by Circular Dichroism (CD) analysis. CD spectra were recorded as previously reported.^[1]^ A Jasco J-715 (Jasco, Tokyo, Japan) spectropolarimeter equipped with a Peltier-type temperature control system (model PTC-348WI) was used. Data are means of two experiments within the indicated ranges (error bars).

**Activities of PPLs with different pesticides**

In the Table S1 is shown the specific activity of the four enzymes used in this study at their optimal temperatures and at 25 °C along with the wavelength at which the assay was followed.^[4]^ The assays were performed with the indicated substrates at the final concentration of 0.5 mM.

| **Table S1 Specific activity of the four PLL enzymes at respective optimal temperatures and at 25 °C with POX, MPOX, MPTON and others OP substrates. n.d.= not detected. Assays were in triplicate. Values are means of two independent experiments within the indicated ranges.** | | | | | | | | | | |
| --- | --- | --- | --- | --- | --- | --- | --- | --- | --- | --- |
|  |  | **Specific activity (U/mg)** | | | | | | | | |
|  |  | ***Sso*Pox** | | ***Sso*PoxW263F** | | ***Sso*3Mut** | | ***Sac*Pox** | | |
| **Substrate** | ** **  **(nm)** | **70 °C** | **25 °C** | **70 °C** | **25 °C** | **65 °C** | **25 °C** | **70 °C** | **25 °C** | |
| **Paraoxon**  **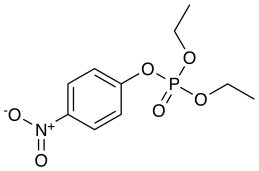** | 405 | 0.40±  0.015 | 0.10±  0.007 | 13.5±  0.6 | 4.8±  0.12 | 31.8±  1.4 | 8.7±  0.4 | 12.03±  0.47 | 1.07±  0.07 | |
| **m-paraoxon**  **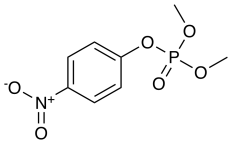** | 405 | 1.20±  0.04 | 0.27±  0.007 | 26.0±  1.2 |  | 31.4±  1.0 | 13.5±  0.7 | 18.53±  1.50 | 6.8±  0.3 | |
| **Parathion**  **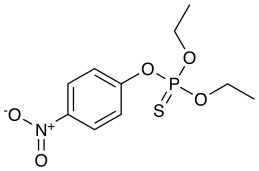** | 405 | 0.004±  0.0002 | n.d. | n.d. | | n.d. | | 0.07±  0.003 | 0.02±  0.00 | |
| **m-parathion**  **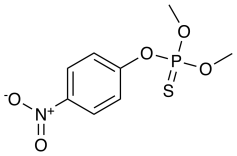** | 405 | 0.017±  0.0005 | 0.003±  0.0001 | n.d. | | n.d. | | 3.07±  0.08 | 0.2±  0.00 | |
| **Diazinon**  **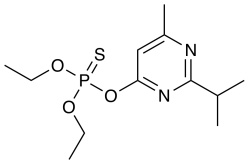** | 228 | 0.011±  0.0010 | 0.044±  0.002 | n.d. | | n.d. | | 0.41±  0.08 | n.d. | |
| **Coumaphos**  **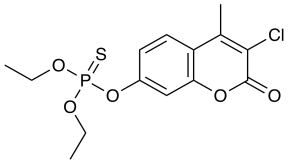** | 348 | 0.002±  0.00015 | n.d. | n.d. | | n.d. | | n.d | n.d. | |
| **Dursban**  **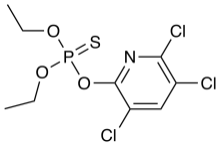** | 276 | 0.0340±  0.0020 | 0.090±  0.007 | .  n.d. | | n.d.  n.d. | | n.d | n.d. | |
| **Malathion**  **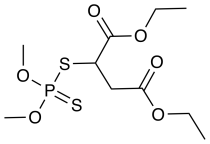** | 405 | 0.011±  0.0001 | 0.005±  0.0003 | n.d. | | n.d. | | n.d. | | n.d. |
| **Phosmet**  **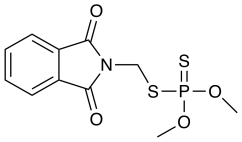** | 405 | 0.006±  0.0004 | 0.001±  0.0004 | n.d. | | n.d. | | n.d. | | n.d. |
| **Dimethoate**  **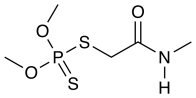** | 405 | 0.010±  0.0007 | 0.002±  0.0001 | n.d. | | n.d. | | n.d. | | n.d. |
| **Tolclophos**  **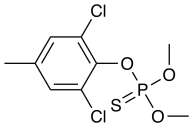** | 224 | n.d. | n.d | n.d. | | n.d. | | n.d. | | n.d. |
| **Cyanophos**  **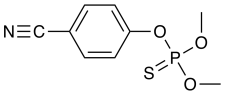** | 246 | n.d. | n.d | n.d. | | n.d. | | 0.096±  0.001 | | n.d. |

The activity was 3-4 fold lower at 25 °C with respect to optimal temperatures. Also mutants that were more active against POX and MPOX had more strict specificity with respect to wt *Sso*Pox and *Sac*Pox. Results reported in the Fig. S2 show clearly the enzymes diversity. These experiments were performed by spotting 500 nmoles of POX on squares (2x2cm) of cotton then submerged in 3 ml of 20 mM Hepes buffer in the presence or not of detergents or organic solvents. In a previous paper we already reported some data on the time-dependent behavior of the four enzymes assayed independently on cotton and glass.^[5]^ Here we have extended incubation times up to 900 min and reported data with 30% ethanol and 30% methanol (v/v) to help extraction from cotton. At indicated times aliquots were removed and read for *p*NP release. The *Sso*3Mut enzyme, at a concentration of 2.5 μg/mL and after only 45 min, was able to remove from cotton almost all of the POX (95%) in a buffered solution and about 80% in the presence of SDS and the commercial soap, but it was dramatically less effective in the presence of ethanol or methanol (5% and 8%, removal). *Sso*W263F mutant achieved results comparable to the triple mutant enzyme (95%) only at 10μg/mL concentration but in the presence of SDS.^[5]^

All enzymes were globally stable to SDS (0.025% w/v), and different degrees of enzyme activation (1-3 fold), already observed in a previous study for *Sso*Pox and *Sso*W263F with respect to the water-only condition,^[5]^ were discovered also in *Sac*Pox and *Sso*3Mut, although the starting activities were different. Furthermore, enzymes showed different profiles of POX degradation over time, specifically in the presence of water-miscible organic solvents ethanol and methanol.

The reason to choose these organic solvents was as to challenge enzymes under particularly stressful conditions being water-miscible solvents extra inactivating to proteins^[6]^ and at the same time hydrophobic enough to help extracting the OPs from surfaces. It is clear that the *Sso*3Mut was more active on shorter time but slightly less stable compared to wild type *Sso*Pox and *Sac*Pox. This behavior is in line with the trade-off between activity and stability typical of thermostable enzymes.^[3]^ Accordingly, it is also worth noting the significant stability of *Sac*Pox in the presence of organic solvents with respect to the other enzymes. The differences in substrate specificity, activity and stability justify the use of a mixture of PLL enzymes as described in detail later.


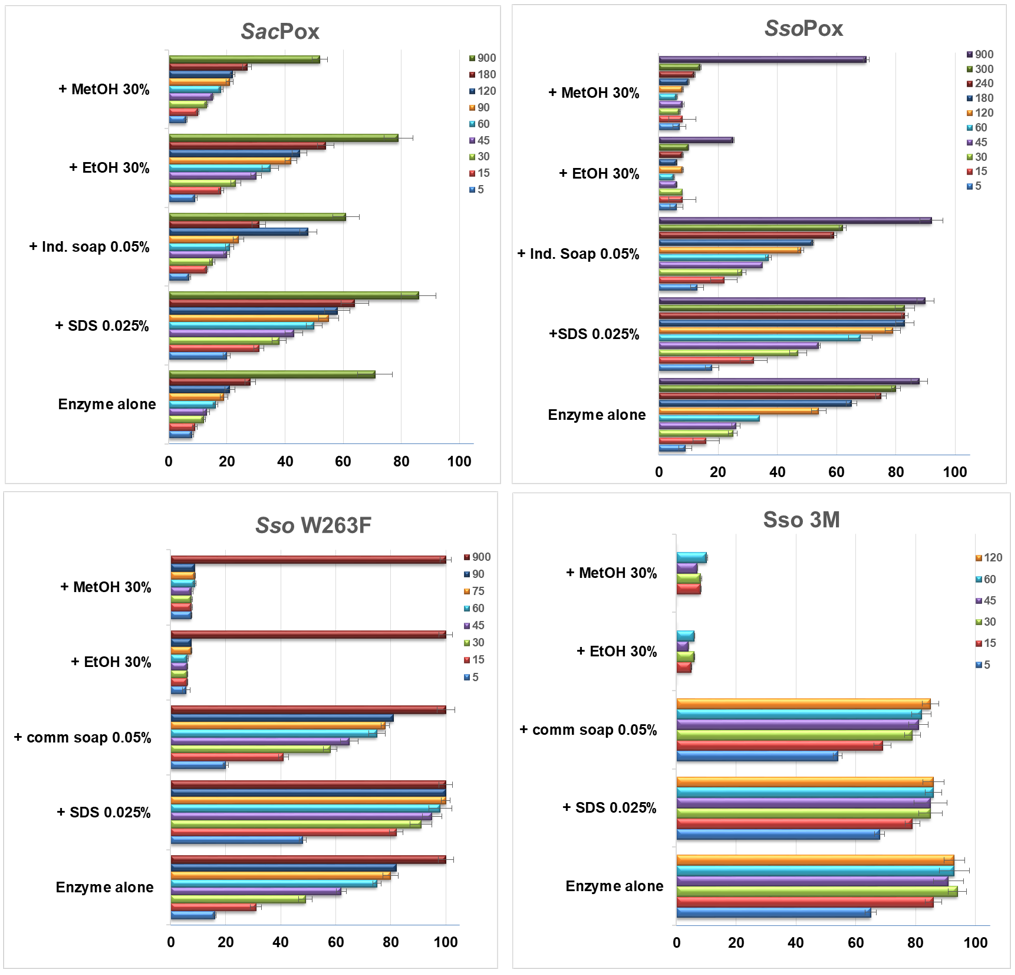


**Figure S2**. Percentages of decontamination of POX (0.5 mM) spotted on cotton tissue (2 x 2 cm) in buffered water solution obtained by using each of the four indicated enzymes (all at 10 μg/ml except *Sso*3Mut at 2.5 μg/ml) in the presence of organic solvent, industrial soap or SDS by incubation at r.t. (21+2 °C) in the range from 5 to 900 minutes. Assays were in triplicate.

Values are means of two independent experiments within the indicated ranges (error bars).

**MEP Optimization parameters**

Preliminary experiments were devoted to select the optimal enzyme substrate for MEP. As reported in Fig. S3 A (i-iv) electroanalytical performances of four common esterase colorimetric substrates [p-nitrophenylbutyrate (pNP-C4), p-nitrophenyhexanoate (pNP-C6), 4-methylumbelliferone (MUB) and 2-naphtyl acetate (NaAc)] were evaluated by cyclic voltammetry (CV). Among the different molecules, only NaAc and MUB could be used. Cyclic Voltammetry (CV) was used to select the best substrate; CVs were recorded from -1.0 to +1.0 V vs. Ag/AgCl, at 25 mV/s, in buffer (20 mM phosphate, pH=7) and 0.1 M KCl in presence of 1mM substrate. An oxidation peak at + 450 mV for NA and at + 620 mV for MUB, respectively was observed. NA was chosen because it gave a stable and reproducible current signal, after addition of the enzyme, in a potential range lower than that of MUB. For the inhibition measurement, DPV was used since it is considered a more sensitive technique than CV. The parameters of DPV were also optimized and the optimal values are: potential range 0 - + 0.9 V, step potential 0.005 V, amplitude 0.07 V, scan rate 0.033V/sec.

In Fig. S3A a-d are reported the data regarding the optimization of the biosensing MEP. The dependence of the response on the amount of the enzyme immobilized on the electrode surface is shown in Fig. S3A a. Because pesticide detection involves an irreversible inhibition of the enzyme, the lowest feasible concentration of enzyme is necessary to reach a low detection limit. The I% value was evaluated towards two different pesticide concentrations of 1 and 5 nM, respectively, for different amount of enzyme (0.25-5.0 U/ml) using 1 and 5 min incubation times with substrate and POX respectively. A value of 1 U/mL was chosen as the best compromise between a low enzyme loading and sufficiently high substrate signal, corresponding to high degree of inhibition.

In Fig. S3B b is reported the optimization of the substrate concentration. Different concentration of NaAc were tested and the current values due to its oxidation evaluated; 0.5 mM was then chosen for the inhibition measurement, given that it was the lowest concentration of substrate still giving the maximum current with high reproducibility, using 1U/mL enzyme, 1 min substrate incubation time and 5 min POX incubation time. In order to decrease the response time, the substrate incubation time and the POX exposure time were evaluated. In Fig. S3B c is reported the behavior of the biosensor towards two different POX concentration, using 1U/mL enzyme, 0.5 mM substrate concentration and 5 sec POX exposure time; a steady-state behavior was obtained after only 30 sec of substrate incubation time, thus this value was chosen for further experiments. Pesticide detection studies were thus carried out at optimized substrate concentration and time (0.5 mM and 30 sec, respectively). In Fig. S3B d is reported the calibration curve for 5 min POX exposure time. The calibration plot was obtained by plotting the values of I % vs. inhibitor concentration and was then fitted by non-linear regression to the four-parameter logistic (4-PL) equation using OriginPro2015 software (Origin Lab Corporation,USA) in which: y is the analytical signal, A is the analytical signal at infinite analyte concentration, B is the analytical signal at zero analyte concentration, x is the analyte concentration, C is the inflection point on the calibration curve (the analyte concentration necessary to have 50 % of the signal, EC50), and *m* is the slope of the linear part of the curve. For 5 min exposure time, a detection limit of 0.4 nM (calculated as the concentration giving 10% inhibition; 0.4 fentomoles in 1 µL of POX solution) ^[7-9]^ was obtained. In comparison to the calibration curve evaluated at 5 sec exposure time (EC50=43 nM), the level of inhibition of the enzyme increased, obtaining a 50% inhibition (EC50) at 1.2 nM. There is always a trade-off between exposure time and sensitivity. However, for this practical analytical application (alarming system) a rapid analysis is recommended. Therefore, an exposure time of 5 sec was used. It is important to underline that this value is lower than most of the data reported in the literature (Table S2). In Fig. S3B e examples of differential pulse voltammograms obtained in absence (dotted line) and in presence of 10 nM POX (solid line) are reported.

**Table S2 Comparison of the analytical performance of enzyme-based electrochemical biosensor for the detection of POX**

| **Enzyme** | **LOD** | **Inhibition time** | **References** |
| --- | --- | --- | --- |
| BChE | 5 ppb | 10 min | [10] |
| BChE | 2 ppb | 30 min | [11] |
| AChE | 5 × 10^−9^ M | 10 min | [12] |
| K42R/K61R EST2 | 0.4 × 10^−9^ M | 5 sec | This work |

A

i ii


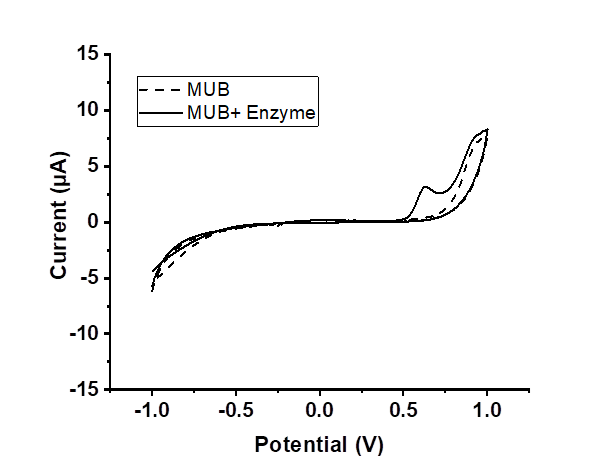

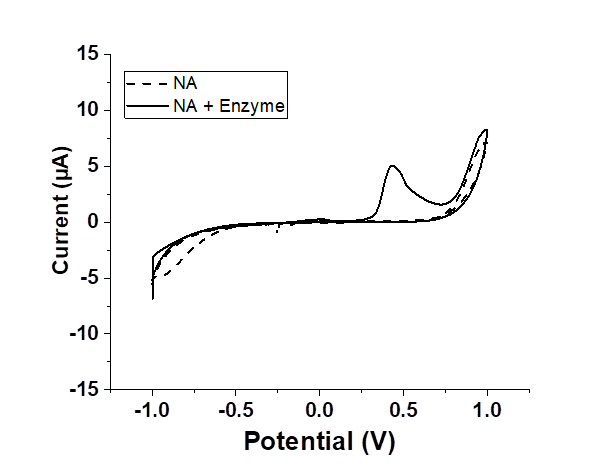


iii iv


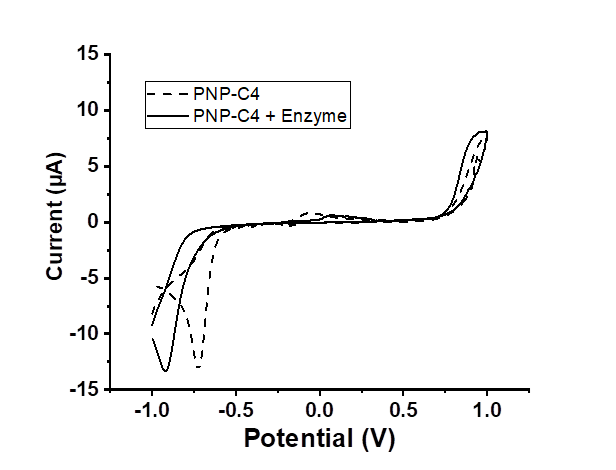

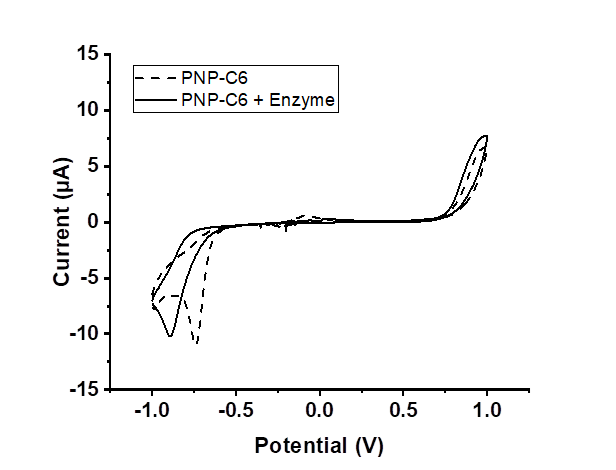


B

a b


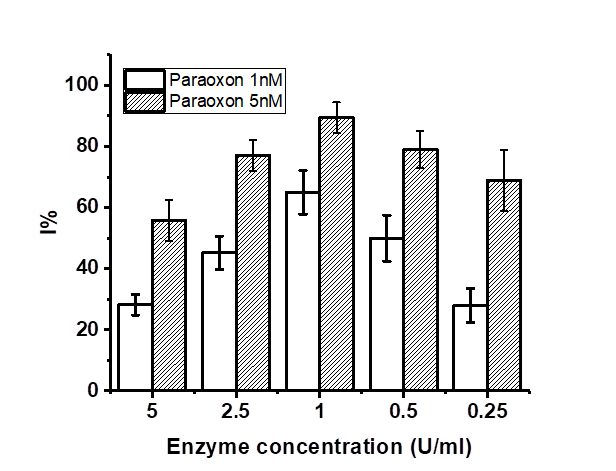

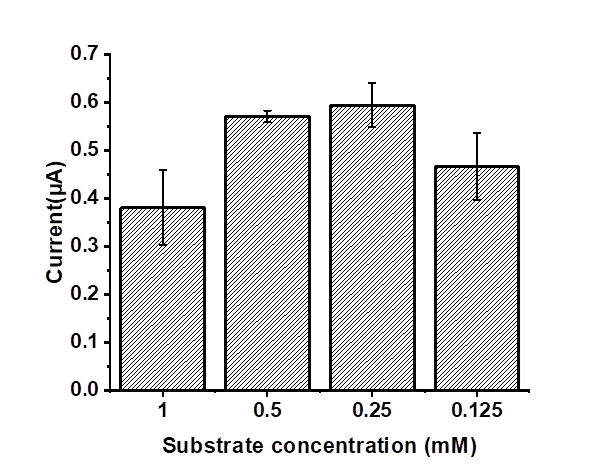


c d


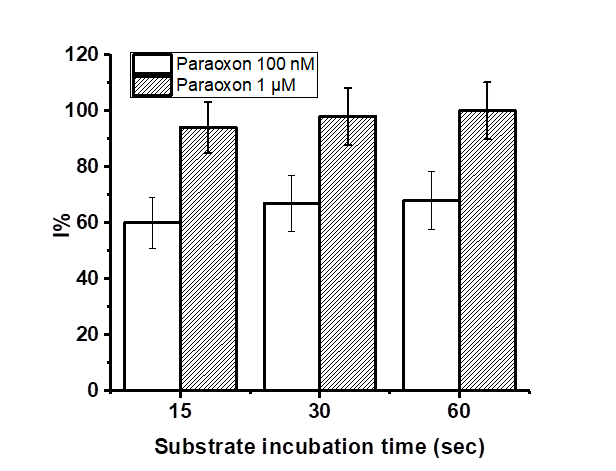

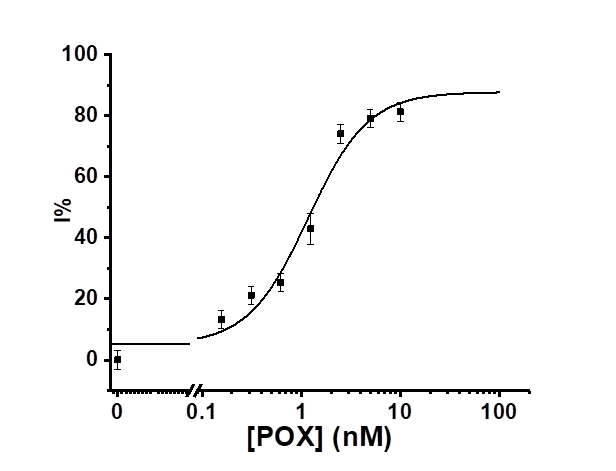


e

**
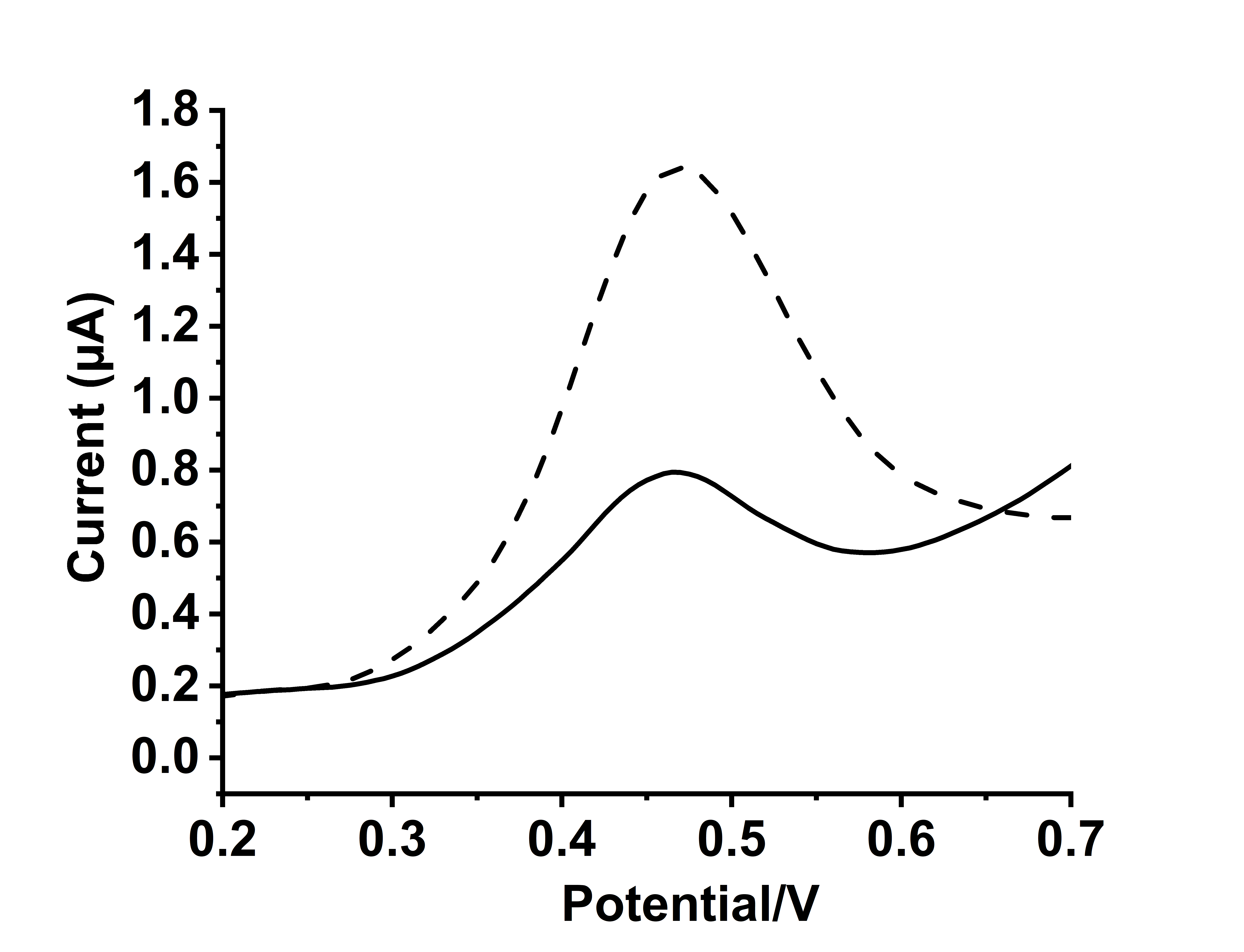
**

**Figure S3.** Optimization experiments of the biosensing MEP; A) Electrochemical characterization of different EST2 substrates using the MEP. The bottom of each well of the platform is screen-printed to form an electrochemical cell, consisting of a graphite WE, a silver RE, and a graphite CE; Cyclic Voltammetry (CV) was used to select the best substrate; CVs were recorded from -1.0 to +1.0 V vs. Ag/AgCl, at 25 mV/s, in 20 mM phosphate buffer (pH=7) and 0.1 M KCl in presence of 1mM substrate (dashed line) and after 15 min of the addition of 5 U/mL of EST2 (solid line). i) CV of NA; ii) CV of MUB; iii) CV of pNP-C4; iv) CV of pNP-C6.; B) Optimization of parameters with NA as substrate. a) Optimization of enzyme concentration using 1 min substrate incubation time and 5 min POX exposure time, for 1 and 5 nM POX respectively; each value is the mean of measurements taken from three experiments, the error bars representing the corresponding s.d.; b) Optimization of substrate concentration using 1 U/mL enzyme, 1 min substrate incubation time and 5 min POX exposure time; each value is the mean of measurements taken from five experiments, the error bars representing the corresponding s.d.; c) Optimization of substrate incubation time using 1U/mL enzyme, 0.5 mM substrate concentration and 5 sec POX exposure time, for 1 and 5 nM POX respectively; each value is the mean of measurements taken from three experiments, the error bars representing the corresponding s.d.; d) Calibration curve of POX using 1U/mL enzyme, 0.5 mM substrate concentration and 5 min POX exposure time. e) Example of differential pulse voltammograms obtained in absence (dotted line) and in presence of 10 nM POX (solid line).

| **Table S2 Four single enzymes with or without SDS were assayed at two different concentrations on NAS substrate. Assays were in duplicate.** | | | | |
| --- | --- | --- | --- | --- |
|  | **Absorbance 405 nm** | | **% hydrolysis** | |
| **Sample** | **Enzyme**  **25 µg/mL** | **Enzyme**  **50 µg/mL** | **Enzyme**  **25 µg/mL** | **Enzyme**  **50 µg/mL** |
| ***Sac*Pox** | 7.5 | 15 | 18 | 37 |
| ***Sac*Pox + SDS** | 6.5 | 18 | 16 | 44 |
| ***Sso*Pox** | 5 | 7 | 12 | 17 |
| ***Sso*Pox + SDS** | 7 | 12 | 17 | 30 |
| ***Sso* Trp263Phe** | 10.5 | 15 | 26 | 37 |
| ***Sso* Trp263Phe + SDS** | 20 | 28 | 49 | 69 |
| ***Sso*3Mut** | 28 | 31 | 69 | 76 |
| ***Sso*3Mut + SDS** | 33 | 32 | 81 | 79 |
| **Blank** | 2.5 | 2.5 | 6 | 6 |
| **Blank + SDS** | 3 | 3 | 7 | 7 |

### Detoxification with free enzymes in water and on selected materials


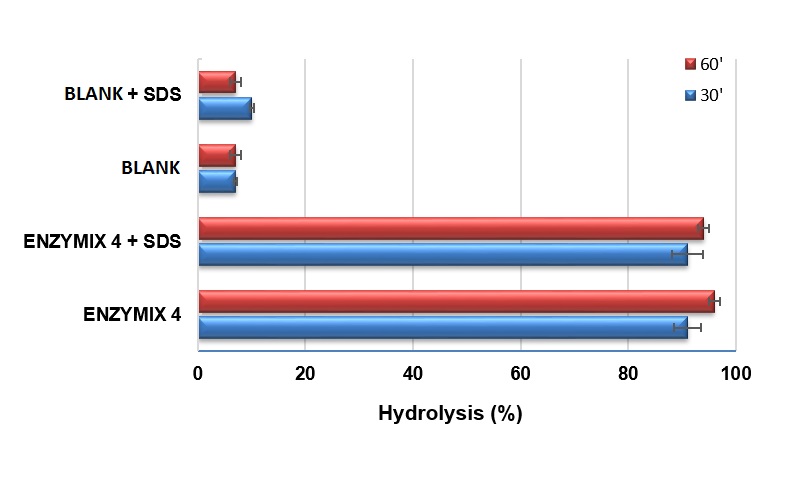


**Figure S4.** Percentages of decontamination of NAS in buffered water solution obtained by using the formulation ENZYMIX4 in the presence or not of SDS (0.025%) by incubation at r.t (21+2 °C) for 30 and 60 minutes. The theoretical end-point of 100% hydrolysis is 40.5 OD based on molar extinction coefficient for the *p*-nitrophenolate ion of 21. Assays were in triplicate**.** Values are means of three independent experiments within the indicated ranges (error bars).


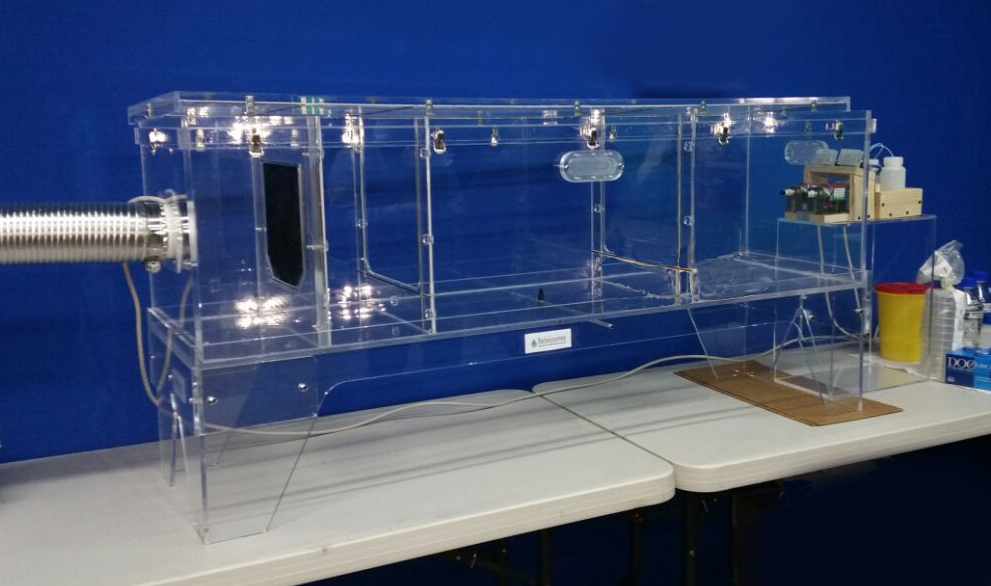


**Figure S5.** Lab bench-size box in which enzymes and substrates were nebulized at the same time.

| **Table S3 Residual *p*-NP after 60 min incubation with ENZYMIX4 was measured spectrophotometrically from the different surfaces. Assays were in triplicate. Experiments were in duplicate and background obtained without enzymes was subtracted. Mean values are shown with indicated ranges (Blk stands for blank). The theoretical 100% degradation was calculated from the extinction coefficient of 21. In a validation experiment made on the same samples by a consultant lab, the amount of *p*-NP was calculated with a calibration curve on HPLC and percentages of degradation compared (Fig. S6).** | | | | | | |
| --- | --- | --- | --- | --- | --- | --- |
| **Sample** | **Abs**  **(405nm)** | **Degradation**  **(%)** | **Mean-Blk**  **(%)** | ***p-*NP**  **(mg/L)** | **Degradation**  **(%)** | **Mean-Blk**  **(%)** |
| **Blk_Aluminium** | 0.0225 ± 0.0025 | 5.5 ± 0.6 |  | 9.84 ± 0.24 | 3.14 ± 0.08 |  |
| **Aluminium** | 0.365 ± 0.012 | 90.1 ± 3.0 | 84.5 ± 3.0 | 229.00 ± 5.00 | 73.1 ± 1.6 | 69.9 ± 1.6 |
| **Aluminium_sds** | 0.384 ± 0.010 | 94.8 ± 2.5 | 89.2 ± 2.5 | 234.12 ± 2.43 | 74.7 ± 0.8 | 71.6 ± 0.8 |
|  |  |  |  |  |  |  |
| **Blk_Linoleum** | 0.010 ± 0.000 | 2.5 ± 0.0 |  | 4.45 ± 0.12 | 1.42 ± 0.04 |  |
| **Linoleum** | 0.127 ± 0.003 | 31.4 ± 0.7 | 28.9 ± 0.7 | 81.65 ± 15.16 | 26.06 ± 4.84 | 24.64 ± 4.84 |
| **Linoleum_sds** | 0.0975 ± 0.007 | 24.1 ± 1.7 | 21.6 ± 1.7 | 74.17 ± 8.73 | 23.67 ± 2.78 | 22.25 ± 2.78 |
|  |  |  |  |  |  |  |
| **Blk_Glass** | 0.030 ± 0.001 | 7.5 ± 0.2 |  | 22.68 ± 0.52 | 7.25 ± 0.17 |  |
| **Glass** | 0.338 ± 0.007 | 83.5 ± 1.7 | 76.0 ± 1.7 | 210.73 ± 0.01 | 67.25 ± 0.003 | 60.00 ± 0.003 |
| **Glass_sds** | 0.315 ± 0.015 | 77.8 ± 3.7 | 70.3 ± 3.7 | 202.78 ± 10.6 | 64.71 ± 3.38 | 57.46 ± 3.38 |
|  |  |  |  |  |  |  |
| **Blk_Cotton** | 0.030 ± 0.010 | 7.5 ± 2.5 |  | 40.19 ± 1.20 | 12.84 ± 0.38 |  |
| **Cotton** | 0.377 ± 0.010 | 93.1 ± 2.5 | 85.6 ± 2.5 | 221.23 ± 0.30 | 70.60 ± 0.10 | 57.76 ± 0.10 |
| **Cotton_sds** | 0.398 ± 0.009 | 98.3 ± 2.3 | 90.8 ± 2.3 | 239.27 ± 0.47 | 76.35 ± 0.15 | 63.51 ± 3.38 |

Considering that the activity of *Sso*3Mut at 25 °C is 8.7 U/mg (see Table **S**1) it is possible to extrapolate that only 23 μl (corresponding to 11 μg) of the original enzyme solution reached the POX solution in the plate, assuming 100% stability of enzyme during the process. The single mutant *Sso*W263F having half the specific activity of *Sso*3Mut at 25 °C (see Table S1), gave a comparable degradation rate considering that it was used at 100 μg/ml, due to its higher affinity for POX.

| **Table S4 Efficiency of decontamination on nebulized samples in the air. In the small box a 100 mL total volume of enzyme or 100 μM POX were nebulized; the nebulization flux was 85 ml/min; enzyme concentration used ranged from 100 micrograms to 510 micrograms per ml; time of incubation for the plate (or materials) was 20 min. Assays were in triplicate. Data are means of two experiments within indicated ranges.** | | | | | |
| --- | --- | --- | --- | --- | --- |
| **Tests** | **Enzymes** | **POX**  **(µM)** | **Conditions** | **Protein used**  **(mg/ml)** | **Degradation**  **(%)** |
| **A1** | *Sso*W263F | 100 | Plate (vol=20ml) | 0.10 | 25 ± 2 |
| **A2** | *Sso*3MUT | 100 | Plate (vol=20ml) | 0.48 | 87.5 ± 5 |
| **B1** | *Sso*3MUT | 100 | Condensed mist | 0.10 | 44 ± 3 |
| **B2** | *Sso*3MUT | 100 | Condensed mist | 0.15 | 44 ± 3 |
| **B3** | *Sso*3MUT | 100 | Condensed mist | 0.20 | 44 ± 5 |
| **B4** | *Sso*3MUT | 100 | Condensed mist | 0.40 | 47 ± 3 |
| **B5** | *Sso*3MUT | 100 | Condensed mist | 0.51 | 50 ± 4 |

From the Table S4 is observed that by increasing the protein concentration up to 510 μg/ml degradation did not increase proportionally. One possible explanation of this behavior is that higher protein concentration affects in some way the coalescence between the POX-containing drops and the enzyme-containing drops. That in turn affects the starting time of the hydrolytic reaction. We did not tested longer time because we were interested to an action as quick as possible starting in the air. This result suggests that for these specific applications highly efficient enzymes are needed. Studies are under way to keep on producing and testing new variants.

**Large-scale Enzyme production**

A large scale manufacturing of *Sso*Pox W263F has been recently described,^[13]^ but so far no one has reported a 150-L scale production of *Sac*Pox and *Sso*Pox3Mut. To reach the aim of a high production of the three enzymes, high cell density fed-batch fermentation experiments were set up on pre-industrial scale coupling a wise design of feeding strategies with alternative induction system based on the use of galactose instead of IPTG.^[13,15]^ Details have been recently reported.^[15]^ The optimized purification process resulted similar for the three enzymes and allowed to reach a total final recovery ranging from 70 to 75% and a pureness grade from 70 to 83% (Table S5). Thus, at the end, a complete manufacturing production and purification process of *Sac*Pox*, Sso*Pox*, Sso*Pox3Mut on large scale drove to a final amount of total units in a range from 155,400 to 453,600 corresponding to a total of 8.9 to 15.1 grams (Table S5). The obtained pureness grades resulted sufficient and compatible with the industrial applications exploited in this research work for the three enzymes and their mixtures.

| **Table S5 Fed-batch fermentations parameters and sample features after downstream processing from recombinant *E. coli* cells. Assays were in triplicate. Data are means of three independent experiments with the indicated ranges.** | | | |
| --- | --- | --- | --- |
|  | ***Strains*** | | |
| **Parameters** | ***E. coli SacPox*** | ***E. coli SsoW263F*** | ***E. coli Sso3Mut*** |
| ***Enzyme recovery (%)** | 75(9) | 72(6) | 70(6) |
| ***Enzyme pureness (%)** | 77(6) | 70(6) | 83(6.5) |
| ***Enzyme total units** | 155,400 (7200) | 253,368(9275) | 453,600(28683) |
| ***Enzyme total grams** | 12.7(0.5) | 8.9(0.4) | 15.1(0.6) |

*****After downstream processing

**
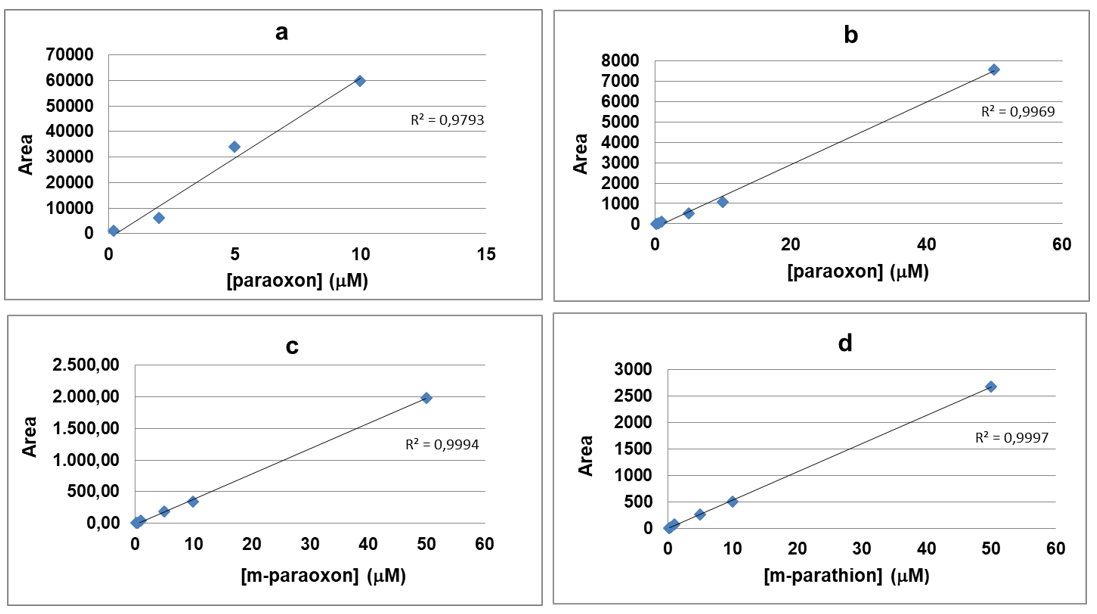
**

**Fig. S6 Calibration curves for a) HPLC (POX) and b-d) GC (POX, MPOX, MPTON, as indicated)**

**Demonstration room description**

As for the small box we designed and built the large demonstration room in the methacrylate material Lexan, but in this case we added a framed internal structure in steel. The chamber remained completely sealed during operations. It was equipped with a sealable door, a non-return air valve, an extractor fan with a filter with activated carbon, a slot for the mounting of the biosensor at half-height of one of the lateral wall and another opening down in a corner for the recovering of the condensed mist. On the ceiling, arriving from the outside, there were two copper pipes ending in two independent rings with three nozzles of 100μm diameter each (see Fig. 2). Two external pumps (MG1 and MG2) were connected through valves to the copper pipes and allowed uniform nebulization of enzyme formulations and NAS at a flow rate of 0.5 L/min.

At the end of the experiment before the door opening, we activated the air aspiration. After recovering of the pieces of materials, the chamber was sealed again and 5M NaOH was nebulized inside in order to eliminate any residual trace of pesticides.

**Theoretical consideration about the sensitivity of biosensor related to the chamber dimension**

The minimum lethal oral dose for Paraoxon/Parathion has been reported to range from 0.17 to 1.471 mg/kg;^[16]^ this oral dose is equivalent to a worker being exposed to about 8 to 69 mg/m^3^ for 30 minutes, assuming a breathing rate of 50 liters per minute and 100% absorption. By using the calibration plot (Fig. 1c), 43 nM (corresponding to 12 mg/m^3^ of POX) is the concentration giving 50% inhibition (EC_50_) for 5 sec exposure time. Thus, considering the above data, the developed biosensing platform possess enough sensitivity to be used as alarming system in the demonstrator chamber. Because we have used 100 μM of NAS in 10m^3^, assuming an even molecular diffusion this should correspond to 0.02 mg/m^3^ namely 600 times lower than EC50 (about 72 picomolar); therefore, in the reality, the NAS solution arrives on the biosensor platform in a more coarse form of 100μm diameter droplets with the original 100 μM concentration.

**References**

1. Pezzullo, M., Del Vecchio, P., Mandrich, L., Nucci, R., Rossi, M., Manco G. Comprehensive analysis of surface charged residues involved in thermal stability in *Alicyclobacillus acidocaldarius* esterase 2. *Prot. Eng. Des. Sel.* **26,** 47-58 (2013).

2. Tokuriki, N., Tawfik, D. S. Stability effects of mutations and protein evolvability. *Current Opinion In Structural Biology* **19**, 596-604 (2009).

3.Tokuriki, N., Stricher, F., Serrano, L., Tawfik, D. S. How Protein Stability and New Functions Trade Off. *PLoS Comput. Biol.* **4***,* 1 (2008).

4. Merone, L., Mandrich, L., Rossi, M., Manco, G. A thermostable phosphotriesterase from the archaeon *Sulfolobus solfataricus*: cloning, overexpression and properties. *Extremophiles,* **9**, 297 -305 (2005).

5. Merone, L., Mandrich, L., Porzio, E., Rossi, M., Müller, S., Reiter, G., Worek, F., Manco G.Improving the promiscuous nerve agent hydrolase activity of a thermostable archaeal lactonase. *Biores. Technol.***, 101**, 9204-9212 (2010**)**.

6. Klibanov, A.M. Improving enzymes by using them in organic solvents. *Nature* **409**, 241-246 (2001**)**.

7. Laschi, S., Ogończyk, D., Palchetti, I., Mascini, M. Evaluation of pesticide-induced acetylcholinesterase inhibition by means of disposable carbon-modified electrochemical biosensors. *Enz. Microb. Techn.***, 40**, 485-489 (2007).

8. Hernandez, S., Palchetti, I., Mascini, M. Determination of anticholinesterase activity for pesticides monitoring using a thiocholine sensor, *Int. J. of Env. Anal. Chem.* **78**, 263(2000).

9. Palchetti, I., Cagnini, A., Del Carlo, M., Coppi, C., Mascini, M., Turner A.P.F. Determination of anticholinesterase pesticides in real samples using a disposable biosensor. *Anal. Chim. Acta* **337**, 315-321 (1997).

10. Arduini, F., Amine, A., Moscone, D., et *al*. Fast, sensitive and cost-effective detection of nerve agents in the gas phase using a portable instrument and an electrochemical biosensor. *Anal Bioanal Chem* **380**, 1049–1057 (2007)

11. [Arduini, F.](http://europepmc.org/search?query=AUTH:%22Arduini+F%22&page=1),  [Ricci, F.](http://europepmc.org/search?query=AUTH:%22Ricci+F%22&page=1),  [Tuta, C.S.](http://europepmc.org/search?query=AUTH:%22Tuta+CS%22&page=1),  [Moscone, D.,](http://europepmc.org/search?query=AUTH:%22Moscone+D%22&page=1) [Amine, A.](http://europepmc.org/search?query=AUTH:%22Amine+A%22&page=1),  [Palleschi, G.](http://europepmc.org/search?query=AUTH:%22Palleschi+G%22&page=1)  Detection of carbamic and organophosphorous pesticides in water samples using a cholinesterase biosensor based on Prussian Blue-modified screen-printed electrode. *Analytica Chimica Acta* **580,** 155–162 (2006).

12. Mishra, R.K., Dominguez, R.B., Bhand, S., Muñoz, R., Marty J.L. A novel automated flow-based biosensor for the determination of organophosphate pesticides in milk. *Biosensors & Bioelectronics* **32**, 56-61 (2012)

13. [Restaino](http://link.springer.com/search?facet-author=%22Odile+Francesca+Restaino%22), O.F., [Baskar](http://link.springer.com/search?facet-author=%22Ujjwal+Bhaskar%22), U., [Paul](http://link.springer.com/search?facet-author=%22Priscilla+Paul%22), P., [Li](http://link.springer.com/search?facet-author=%22Lingyun+Li%22), L., [De Rosa](http://link.springer.com/search?facet-author=%22Mario+De+Rosa%22), M., [Dordick](http://link.springer.com/search?facet-author=%22Jonathan+S.+Dordick%22), J.S., [Linhardt](http://link.springer.com/search?facet-author=%22Robert+J.+Linhardt%22), R.J. High cell density cultivation of a recombinant E. coli strain expressing a key enzyme in bioengineered heparin production. *Appl. Microbiol. Biotechnol.***, 97**, 3893-3900 (2013).

14. Restaino, O.F., Borzacchiello, M.G., Scognamiglio, I., Porzio, E., Manco, G., Fedele, L., Donatiello, C., De Rosa, M., Schiraldi, C. Boosted large-scale production and purification of a thermostable archaeal phosphotriesterase-like lactonase for organophosphate decontamination. *J. Ind. Microbiol. Biotechnol*.**, 44**, 363-375 (2017).

15. Restaino, O.F., Cimini, D., De Rosa, M., Catapano, A., De Rosa, M., Schiraldi, C. High cell density cultivation of Escherichia coli K4 in a microfiltration bioreactor: a step towards improvement of chondroitin precursor production. *Microbial. Cell Fact.* **10**, 10 (2011).

16. NIOSH [2013]. Current intelligence bulletin 66: derivation of immediately dangerous to life or health (IDLH) values. Cincinnati, OH: US Department of Health and Human Services, Centers for Disease Control and Prevention, National Institute for Occupational Safety and Health, DHHS (NIOSH) Publication 2014–100.
